# The Evolution of the Discrete Multirenculate Kidney in Mammals from Ecological and Molecular Perspectives

**DOI:** 10.1093/gbe/evad075

**Published:** 2023-05-09

**Authors:** Xu Zhou, Wenqi Rong, Boxiong Guo, Xiaofang He, Li Cao, Yu Zheng, Shixia Xu, Guang Yang, Wenhua Ren

**Affiliations:** Jiangsu Key Laboratory for Biodiversity and Biotechnology, College of Life Sciences, Nanjing Normal University, Nanjing, China; Jiangsu Key Laboratory for Biodiversity and Biotechnology, College of Life Sciences, Nanjing Normal University, Nanjing, China; Jiangsu Key Laboratory for Biodiversity and Biotechnology, College of Life Sciences, Nanjing Normal University, Nanjing, China; Jiangsu Key Laboratory for Biodiversity and Biotechnology, College of Life Sciences, Nanjing Normal University, Nanjing, China; Jiangsu Key Laboratory for Biodiversity and Biotechnology, College of Life Sciences, Nanjing Normal University, Nanjing, China; Jiangsu Key Laboratory for Biodiversity and Biotechnology, College of Life Sciences, Nanjing Normal University, Nanjing, China; Jiangsu Key Laboratory for Biodiversity and Biotechnology, College of Life Sciences, Nanjing Normal University, Nanjing, China; Jiangsu Key Laboratory for Biodiversity and Biotechnology, College of Life Sciences, Nanjing Normal University, Nanjing, China; Jiangsu Key Laboratory for Biodiversity and Biotechnology, College of Life Sciences, Nanjing Normal University, Nanjing, China

**Keywords:** mammals, origin, discrete multirenculate kidney, genes related to duplex kidney, convergent evolution

## Abstract

Mammals have developed different kinds of renal structures during evolution, yet the origin of the renal structural phenotypes and the molecular mechanisms underlying their adaptive evolution remains unclear. Here, we reconstructed the ancestral state of the renal structures across mammals and found that the unilobar kidney was the ancestral character in mammals. The subsequent correlation analyses between renal phenotypes and life history traits revealed that species with a larger body or in aquatic habitats tend to have evolved discrete multirenculate kidneys (DMKs). To explore the molecular convergent mechanisms among mammals with this most distinct renal structure, the DMK, we used 45 genes related to duplex/multiplex kidney diseases to compare the evolutions of species with DMKs and with other renal phenotypes. Twelve rapidly evolving genes that were functionally enriched in cilium assembly and centrosome were identified in species with DMKs, suggesting that these genes played key roles in the evolution of DMKs. In addition, positive selection was detected in six crucial genes which are mainly involved in epithelial tube morphogenesis and the regulation of neurogenesis. Finally, 12 convergent amino acid substitutions, 6 of which are in crucial domain of proteins, were shared by 2 or more lineages with DMKs. These findings could provide some novel insights into the origin and evolution of renal structures across mammals and the pathogenesis of renal diseases in humans.

**Significance**

Previously, the origin and evolution of renal structures in mammals remain indistinct. Herein, the ancestral state reconstruction and the association analyses in 62 species were performed to uncover the evolutionary history of renal structures in mammals and the driving factors in it, respectively. Also, the comparative analyses of 45 genes related to duplex kidney in 41 species were executed to elucidate the convergent evolution among mammals with multirenculate kidney. Besides, the crucial sites or genes in this study may feed back into human renal diseases.

## Introduction

The kidney is an essential metabolic organ in mammals and looks like a reddish-brown bean. Its basic function is to produce urine and clear the system of metabolic waste and poisons (Finco 1997). A whole kidney consists of renal parenchyma, which is a combination of the outer renal cortex and the inner renal medulla and renal pelvis (Rouiller 1969). The renal cortex is composed of glomerulus, partial renal tubules, blood vessels, and cortical collecting ducts (Kriz and Bankir 1988). Ultrafiltration occurs in the renal cortex, which also produces erythropoietin. Another part of the renal parenchyma, the renal medulla, contains the loop of Henle, medullary capillary plexus, vasa rectae, and more (Kriz 1981). Its function is to maintain the balance of salt and water in blood and reabsorb water. In addition, the renal pelvis acts as the funnel through which urine flows into the ureter (Schmidt–Nielsen 1987; Dwyer and Schmidt-Nielsen 2003; Marieb and Hoehn 2006).

So far, two basic renal structural types have been found in mammals: 1) the unilobar kidney (UK) with continuous cortex and medulla and 2) the multilobar kidney with continuous/discrete cortex and discrete medulla (Oliver 1968). The unipapillary kidney, the simpler renal type in mammals, is made up of renculi, which consist of the renal cortex, renal medulla, and renal pelvis (Hodson 1972). There are actually two other types of renal structures—a crest kidney and a kidney with tubi maximi—that are simply enlargements of the renculus unit (Kriz et al. 2008). What is more, multilobar kidneys, which are made of multiple renculus units, can be divided into CMKs and discrete multirenculate kidneys (DMKs) according to whether the cortex is compound or discrete (Jamison and Kriz 1982; Dantzler 1989). Interestingly, the unipapillary kidney is found mainly in small species, like mice and cats; the crest kidney in, for example, monkeys and camels; and the kidney with tubi maximi in, for example, horses and hippopotamuses. Finally, the CMK is found in humans, pigs, beavers, and manatees and the DMK in cetaceans, pinnipeds, bears, elephants, and more (Beuchat 1996).

Over the past few decades, numerous hypotheses or conjectures about the origin and evolution of renal structures in mammals have been proposed. The UK may be the original renal structure in mammals, and then, the complex multilobar kidney appears to be derived from it (Verhaegen 1993; Beuchat 2002). Almost all marine mammals have multilobar kidneys, and the majority of nonmarine aquatic mammals may have inherited the multilobar kidney phenotype from their ancestors that lived in marine environments (Ortiz 2001). Nevertheless, terrestrial mammals with lobed kidneys (e.g., bears, elephants, and rhinos) may also have inherited multilobar kidneys from their semiaquatic ancestors (Lavergne et al. 1996; Gaeth et al. 1999). For external or internal structures, the DMK is clearly the most distinct one. The mammalian DMK was thought to have originated as an adaptive response to a large body size, deep and prolonged diving in aquatic environments, and a hypertonic marine diet (Williams 2006). Because marine mammals live in a hypertonic environment, which increases intracellular dehydration if saline water is ingested (Williams 1997), the lobulated state of the marine mammal kidney seems to be an adaptation, because the enlarged surface area between the renal cortex and renal medulla can enhance its ability to rapidly handle hypertonic fluids, excrete excess salt and nitrogenous wastes, and relieve saline-induced intracellular dehydration (Williams 2006). The high filtration rate of the glomeruli allows the kidneys of marine mammals to operate at a high energy rate between dives. In addition, the kidneys of marine mammals can resist the effects of reduced blood supply during diving (Pfeiffer 1997). To accommodate the absolute increase in metabolism and the corresponding increase in excretion of end products with increasing body size, the number of nephrons required increases. However, as the number of nephrons required exceeds the maximum number that can be contained in an unipapillary kidney, the unipapillary kidney may give way to the multilobed kidney. Although these suppositions and reports provide us with considerable insights into the origin and evolution of multilobar kidneys in mammals, more evidence is needed to support them. Furthermore, the molecular evidence underlying the phenotypic convergence among species with DMKs remains unknown.

In this study, we reconstructed the ancestral state of renal structures in mammals and explored the factors that drove the evolution of DMKs in mammals. Then, we analyzed genes involved in duplex/multiplex kidney formation to reveal the molecular convergent mechanism among mammals with DMKs from aspects of rapid evolution, positive selection, and convergent amino acid substitution. Our study may also provide implications for the etiology and genetic mechanisms underlying duplex/multiplex kidney diseases in human.

## Results

### UK as the Ancestral State of the Mammalian Renal Structure

**Fig. 1.**
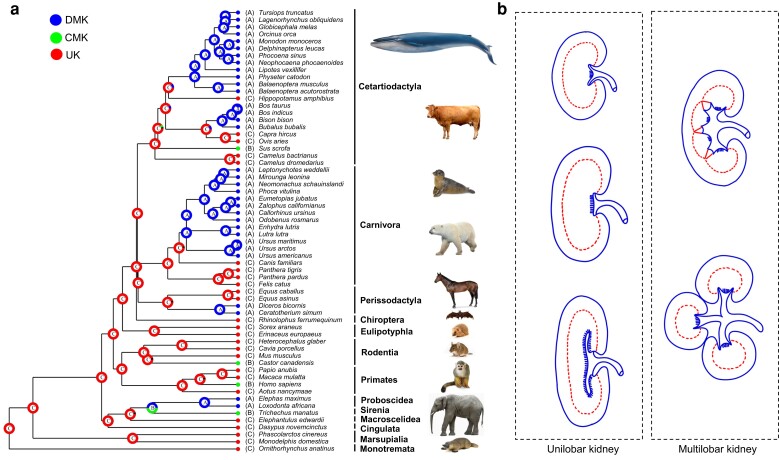
Evolution of renal structures across mammals. (*a*) Ancestral state reconstruction of the renal structures in mammals. Pie diagrams at each nodes indicate proportional likelihoods of each state, with UK, CMK, and DMK. Ancestral character states of the renal structure were reconstructed using the DEC + J model with RASP software. The images of representative species were downloaded from an image search engine (https://yandex.com/images/). (*b*) The display of the vertical section of different kinds of renal structures. The UKs on the left, from top to bottom, are unipapillary kidney, crest kidney, and the kidney with tubi maximi; multilobar kidneys on the right, from top to bottom, are CMK and DMK. The pictures were depicted according to the literature from Charles R in 1969 (Rouiller 1969).

We mapped the phenotypic states of renal structural types onto the species tree from TimeTree (Kumar et al. 2017) (supplementary fig. S1, Supplementary Material online). The evolution of the renal structure across the mammalian phylogeny is summarized graphically in figure 1. Intuitively, there are five finely sorted renal structures in mammals (fig. 1*b*). The ancestral state reconstruction showed that the renal structural phenotypes are diverse among mammals (fig. 1*a* and supplementary table S1, Supplementary Material online). By comparing the results generated from six models applied for reconstruction, we found that the best-fit model was the DEC + J one, which is derived from the DEC model and specifies the weight of each jump dispersal event in the cladogenesis matrix by assigning a parameter—j (Matzke 2014) (supplementary table S2, Supplementary Material online). The reconstruction with the DEC + J model supported that the UK, whose fit probability is 0.9919 in the ancestral node of whole mammals, is the ancestral state for mammals and revealed that the multilobar kidney phenotype is derived from it multiple times in several independent lineages (fig. 1*a* and supplementary table S3, Supplementary Material online). What is more, the result suggested that renal structural phenotypes shifted from the UK to the DMK in the ancestral node of Proboscidea and some internal nodes within Cetartiodactyla, Carnivora, and Perissodactyla. In addition, the UK evolved into the CMK in the ancestral node of Sirenia, and some internal nodes within Primates, Rodentia, and Cetartiodactyla (fig. 1*a*).

### Relationships between Life History Traits and Renal Structural Types

Because life history traits here are classified into continuous and discrete types (supplementary table S1, Supplementary Material online), we used different tools with specific and best-fitting models to detect the correlation between life history traits and renal structural types in mammals. First, we utilized Caper, an R package, to analyze the relationship between body size and renal structure types with the phylogenetic generalized least squares (PGLS) method, which showed a significant association between two characters, as expected (*R*^2^ = 0.1055, *P* < 0.05, *n* = 62) (table 1). Then, we assessed the correlations between the renal structural types and two discrete traits in the phylolm package with a phylogenetic logistic regression method, which revealed a significant correlation between the renal structure type and habitat (*P* < 0.05, *n* = 62) but did not detect any association between the renal structure type and diet (table 1).

**Table 1:** Correlation Test between Structural Kidney Types and Life History Traits Using Different Kinds of Methods Based on Phylogeny

Methods	Tools	Predictors	Parameters in results
Phylogenetic generalized least squares	Caper (R package)	Body mass	0.1055 (*R*^2^); 1 (*λ*); −1.4914(AIC); 0.0058 (*P* value)
Phylogenetic logistic regression	phylolm (R package)	Habitat	0.0120 (alpha); 0.6621 (standard error); 0.0071 (*P* value)
		Diet	0.0085 (alpha); 0.7849 (standard error); 0.2677 (*P* value)

*λ* (lambda) represents phylogenetic signal; alpha is the phylogenetic correlation parameter estimate. Analyses were conducted using the tree downloaded from TimeTree, and the method is from R package “Caper” and “phylolm.” The standard error for each alpha estimate is also given.

Given that habitat was significantly correlated with the evolution of renal structures, we then searched for dependent relationships between these variables. The results showed that habitat was dependent on renal structures or both traits were dependent on each other, but renal structures were not shown to be dependent on habitat (table 2).

**Table 2:** Results of Likelihood Analyses of Correlated Evolution between Renal Structures and Habitats

Dependent variable	Independent model (AIC)	Dependent model (AIC)	Likelihood ratio	*P* value
Renal structures	108.2251	107.7110	4.5142	0.1047
Habitats	108.2251	102.0261	10.1990	0.0061
Renal structures and habitats	108.2251	105.1715	11.0537	0.0260

The likelihood ratio analysis was based on best-fitting model “fitMk” with R package “phytools.” Three dependent models were tested: (*a*) renal structures depend on habitats, (*b*) habitats depend on renal structures, and (*c*) both traits depend on each other.

### Selection Tests of Genes Related to Duplex Kidney Formation across Mammals

Based on the consensus tree from TimeTree (supplementary fig. S2, Supplementary Material online), we used the branch model in Codeml of PAML to identify rapidly evolving genes (REGs) in 41 mammals, which were divided into a DMK group and a nondiscrete multirenculate kidney (non-DMK) group. The results revealed that divergent selective pressure might have acted on mammals with different kinds of renal structures.

**Fig. 2.**
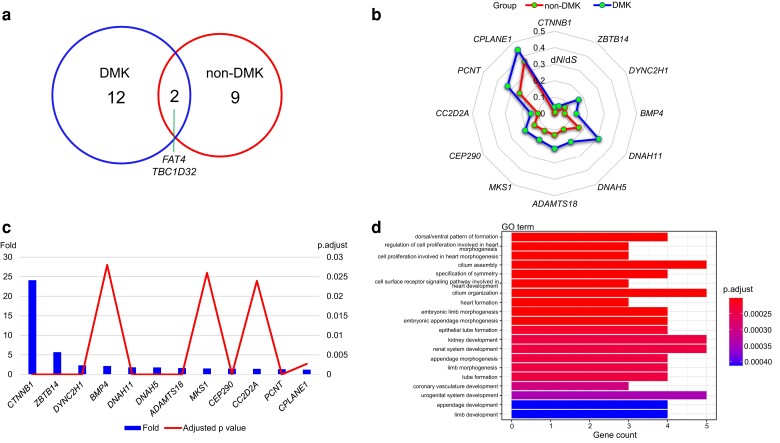
REGs in DMK and non-DMK mammals. (*a*) DMK species-specific REGs, non-DMK species-specific REGs, and the overlapping REGs in both groups. (*b*) *d*_N_/*d*_S_ values of 12 REGs in DMK species are compared with non-DMK species (corrected by FDR, *P* < 0.05). (*c*) The value of *ω*_DMK_/*ω*_non-DMK_ of 12 REGs combined with adjusted *P* value in DMK mammals. (*d*) GO enrichment of REGs in DMK mammals. Top 20 terms in the biological process are shown. DMK: discrete multirenculate kidney; non-DMK: non-discrete multirenculate kidney.

After correcting for multiple testing by false discovery rate (FDR, adjusted *P* < 0.05), we identified 14 REGs in DMK species, 11 REGs in non-DMK species, and 2 genes (i.e., *FAT4* and *TBC1D32*) that overlapped in both groups (fig. 2*a*, supplementary table S4 and S5, Supplementary Material online). The result also suggested that the evolutionary rates of these DMK-specific REGs are significantly greater than those of non-DMK species, as high as 24.1-fold (fig. 2*b* and *c*). In addition, in light of gene counts and significance, the Gene Ontology (GO) enrichment analysis showed that these DMK species-specific REGs are significantly enriched for several biological processes, such as cilium assembly, kidney development, urogenital system development, and the cellular components cilia and centrosome (fig. 2*d* and supplementary table S6, Supplementary Material online). The Kyoto Encyclopedia of Genes and Genomes (KEGG) enrichment analysis suggested that these REGs were overrepresented in several KEGG pathways associated with neurodegeneration and regulating the pluripotency of stem cells (supplementary table S7, Supplementary Material online). However, enrichment analysis of REGs unique to non-DMK species showed no significant enrichment results, which may be due to unconcentrated functions of these genes.

With the branch–site model, fifteen positively selected sites in six key genes were identified in DMK species, and functional enrichment showed that these genes are associated with epithelial tube morphogenesis and regulation of neurogenesis (biological process [BP]), proteoglycan binding (molecular function [MF]), and axon guidance (KEGG) (table 3 and supplementary table S8 and fig. S3, Supplementary Material online). To further explore whether the positive selection of these sites is related to the function of the protein, we searched the UniProt database and mapped the positively selected sites onto 3D structures of the corresponding proteins, the results showed that several sites are in crucial functional domains of proteins (e.g., CTNNB1 protein, site 167, ARM domain; ROBO2 protein, site 597, fn3 domain) (table 3 and supplementary fig. S4, Supplementary Material online). Furthermore, 66.6% (10/15) of the positively selected sites were found to have undergone radical amino acid property changes (table 3).

**Table 3:** Positively Selected Genes and Sites in DMK Mammals Detected by the Branch–Site Model

Gene	2ΔlnL	Model	*ω* value	Adjusted *P* value	Positively selected sites	TreeSAAP
*ROBO2*	5.7352	Ma	*ω*0=0.0365, *ω*1=1.0000, *ω*2=1.6401	0.0166	** 597(0.894-fn3)**	*P_α_ P_c_ P*
		Ma0	*ω*0=0.0363, *ω*1=1.0000, *ω*2=1.0000		1006(0.996^b^)	
*PTPRS*	21.5826	Ma	*ω*0=0.0189, *ω*1=1.0000, *ω*2=42.3625	<0.0001	736(0.929)	
		Ma0	*ω*0=0.0184, *ω*1=1.0000, *ω*2=1.0000		1051(0.984^a^)	*pHi*
*SLIT2*	7.9534	Ma	*ω*0=0.0296, *ω*1=1.0000, *ω*2=1.2290	0.0095	**279(0.922-LRRNT)**	*P_α_*
		Ma0	*ω*0=0.0296, *ω*1=1.0000, *ω*2=1.0000		**984(0.893-EGF)**	*P_r_ α_c_*
*PLXNB2*	6.5452	Ma	*ω*0=0.0344, *ω*1=1.0000, *ω*2=55.5446	0.0158	263(0.813)	
					1099(0.829)	*K^0^ P_r_ P_α_ P_c_ α_n_ P*
		Ma0	*ω*0=0.0341, *ω*1=1.0000, *ω*2=1.0000		1371(0.994^b^)	*pK’*
*CTNNB1*	8.2208	Ma	*ω*0=0.0016, *ω*1=1.0000, *ω*2=3.6606	0.0096	123(0.994^b^)	
		Ma0	*ω*0=0.0016, *ω*1=1.0000, *ω*2=1.0000		**167(0.974^a^-ARM)**	*N_s_ P_β_ B_l_ P_c_ F R_a_ H_p_ P*
*MKS1*	5.9156	Ma	*ω*0=0.0825, *ω*1=1.0000, *ω*2=1.1461	0.0151	191(0.905)	
					**261(0.890-B9-C2)**	*pHi*
		Ma0	*ω*0=0.0824, *ω*1=1.0000, *ω*2=1.0000		**364(0.864-B9-C2)**	*P_α_*
					405(0.900)	*R_F_ P_c_ F R_a_ H_t_ P*

The key functional domains with positively selected sites are shown in bold font in parentheses. Twenty-one amino acid properties in TreeSAAP: *P_α_*, α-helical tendencies; *N_s_*, average number of surrounding residues; *P_β_*, β-structure tendencies; *B_l_*, bulkiness; *B_r_*, buriedness; *R_F_*, chromatographic index; *P_c_*, coil tendencies; *c*, composition; *K^0^*, compressibility; *pK’*, equilibrium constant for ionization of COOH; *C_a_*, helical contact energy; *h*, hydropathy; *pHi*, isoelectric point; *E_l_*, long-range nonbonded energy; *F*, Mean r.m.s. fluctuational displacement; *M_v_*, molecular volume; *M_w_*, molecular weight; *H_nc_*, normalized consensus hydrophobicity; *V^0^*, partial specific volume; *P_r_*, polar requirement; *P*, polarity; *α_c_*, power to be-C-term of the *α*-helix; *α_m_*, power to be-middle of the *α*-helix; *α_n_*, power to be-N-term of the *α*-helix; *μ*, refractive index; *E_sm_*, short- and medium-range nonbonded energy; *R_a_*, solvent accessible reduction ratio; *H_p_*, surrounding hydrophobicity; *H_t_*, thermodynamic transfer hydrophobicity; *E_t_*, total nonbonded energy; *P*, turn tendencies.

a Posterior probabilities (pp) of BEB > 95%.

b pp > 99%.

### Convergent Amino Acid Substitutions among Species with DMKs

**Fig. 3.**
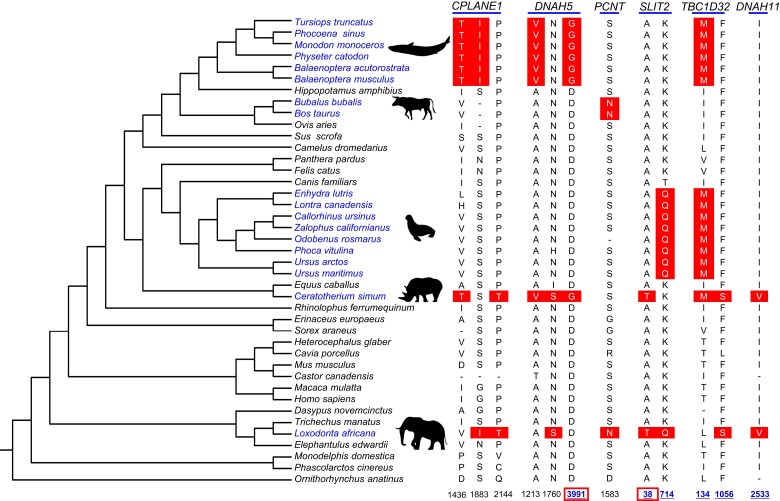
Convergent amino acid substitutions among DMK mammals. The figure showed the DMK group, which are classified into five taxa. The convergent amino acid substitutions are clearly shown in the picture. The number of the sites in key functional domains is marked clearly too, combined with underlines and bold font, although the one with a box means the mutation in this site of the corresponding protein may be deleterious or damaging.

Twelve group-specific convergent amino acid changes were determined in six proteins: CPLANE1 (V1436T, S1883I, and P2144T), DNAH5 (A1213V, D3991G, and N1760S), PCNT (S1583N), SLIT2 (K714Q, A38T), TBC1D32 (F1056S, I134M), and DNAH11 (I2533V). Among these sites, TBC1D32 (I134M) was shared by three lineages with a DMK (Cetacea, aquatic Carnivora, and rhinoceros in Perissodactyla), and elephants shared five out of all convergent amino acid substitutions with rhinoceros in five genes (fig. 3).

Further exploration on how these convergent amino acid changes affect protein function found that six convergent amino acid changes were in the key domain of proteins (e.g., DNAH5 protein, site 3991, Dynein heavy chain region D6 P-loop domain; SLIT2 protein, site 714, site 38, EGF-like domain; TBC1D32 protein, site 134, site 1056, BROMI domain; and DNAH11 protein, site 2533, AAA^+^ lid domain) (fig. 3). Meanwhile, we detected several amino acid changes that may be deleterious or damaging when the protein is functioning (DNAH5 protein, site 3991; SLIT2 protein, site 38) (fig. 3). Among them, the site 3991 of the DNAH5 protein changed from an acidic, negatively charged aspartic acid (D) to a polar, uncharged glycine (G); the site 38 of SLIT2 protein, too, changed from a nonpolar, hydrophobic alanine (A) to polar, uncharged, hydroxyl-containing threonine (T).

## Discussion

### The Multilobar Kidney Independently Evolved Multiple Times in Mammals

Until now, researchers offered many hypotheses about the origin of renal structures in mammals. The lobulation of the kidney in marine mammals was thought to excrete excess salt and nitrogen waste by expanding the surface area between the medulla and the outer cortex (Williams 2006). However, several lineages of freshwater aquatic mammals and terrestrial mammals shared similar renal structures with marine mammals, and they were considered to have inherited the multilobar kidney from ancestors who originally inhabited marine environments and semiaquatic marine ancestors (Domning 2001; Wursig and Perrin 2009). Previous studies also suggested that the simple UK may be primitive for mammals although the complex multilobar kidney may have derived from it (Straus and Arcadia 1958; Verhaegen 1993). Inferring ancestral states can suggest the evolutionary trajectory of a trait by mapping various phenotypes of living taxa onto phylogenies (Bokma 2008).

Our ancestral state reconstruction suggested that the UK existed in several deep nodes of the mammalian phylogeny, which confirms that the UK is the primordial phenotype of mammals from which the complex multilobar kidney evolved (fig. 1*a*). Multiple lineages were inferred to have derived multilobar kidneys (e.g., Cetartiodactyla, Carnivora, Perissodactyla, Rodentia, Primates, Proboscidea, and Sirenia). In more details, CMKs were derived in some lineages (e.g., Cetartiodactyla, Rodentia, Primates, and Sirenia), although DMKs were inferred to occur in several lineages (e.g., Cetartiodactyla, Carnivora, Perissodactyla, and Proboscidea). In summary, our reconstruction of the evolutionary trajectory of the renal structure 1) was based on a widely accepted phylogeny and covered nearly all orders of mammals and 2) adds more reliable information about the origin and evolution of the renal structure. What is more, this finding on the independent evolution of the multilobar kidney may have intriguing implications for research on convergent phenomena.

### The Evolution of Renal Structures Was Driven by Body Size and Habitats in Mammals

Species have evolved various life histories based on their environments, and some individual species have evolved adjustable life histories to physiologically respond to different environments (Fisher 1958; Scheiner 1992). Over the past few decades, a lot of correlation analyses have been conducted on renal phenotypes and other traits in mammals and vertebrates. For example, body weight was found to be positively correlated with kidney weight and renal blood flow (Prothero 1984), whereas body mass was positively correlated with cortical thickness and total/outer/inner medullary thickness and negatively correlated with relative medullary thickness and maximum urine osmolality, and maximum urine osmolality was positively correlated with relative medullary thickness (Beuchat 1996). Accordingly, body mass seems important to various renal phenotypes.

Here, we found significant correlations between renal structure and two traits (body mass and habitats) based on the consensus phylogeny, which confirmed the two previous hypotheses about the evolution of renal structures in mammals (King 1991) (table 1). The inference mentioned above explains the correlation between renal structure and body mass: to adapt to the absolute increase in metabolism needed for an increase of body size and the number of needed nephrons increased beyond the optimal nephron number of UKs, and thus, the multilobar kidney emerged (Dantzler 1989). Thus, mammals with larger body sizes tend to evolved DMKs.

The change from UK to multilobar kidney may be related to the physical force required to move the fluid along the nephron and the need to reabsorb filtered solutes and water (Dantzler and Braun 1980; Calder and Braun 1983). Previous studies revealed a correlation between glomerular development and habitat in vertebrates; meanwhile, glomeruli are structurally located in the renal cortex (Marshall and Smith 1930). In short, these signs support the finding in our study (table 2) that there is a mutual dependent relationship between the renal structure and habitats. Therefore, aquatic mammals tended to derive DMKs. Understanding the origin and evolution of renal structures across mammals is vital and can make it easier to explore the molecular mechanisms underlying the adaptive evolution of mammals.

### Potential Molecular Mechanisms of Convergent Evolution among Mammals with DMKs

Convergent evolution occurs when species that live in the same or similar environments for a long time evolve similar phenotypes. Convergent phenotypic evolution provides a unique perspective on how the genome encodes phenotypes. Recently, many studies have shown that the existence of phenotypic convergence can be seen at different molecular levels, such as convergent amino acid substitutions, convergent REGs, convergent positively selected genes, and convergent changes in amino acid bias (Pascoal et al. 2014; Foote et al. 2015; Hao et al. 2019).

Physiological adaptation or adaptive characters are a homeostatic mechanism in response to instant environmental stimuli, whereas evolutionary adaptation is the result of reproductive success reflecting natural selection (Bennett 1997). The mammalian kidney is critical for homeostasis because it plays a major role in the excretion of metabolic waste, the regulation of extracellular fluid volume, electrolyte balance, and acid–base balance (Berglund et al. 2020). Crucial factors in the evolution of the renal structure and function of the vertebrate appear to be related to body fluid regulation, including the maintenance of constant water and salt levels in the body (Mahasen 2016). In the present study, we found several possible molecular mechanisms that account for the phenotypic convergence of DMK mammals.

First, 12 genes involved in duplex/multiplex kidney formation were found to have evolved rapidly only in DMK mammals, and the functional enrichment analyses of these genes were mainly about cilium assembly, kidney development, urogenital system development, and two cellular components (cilia and centrosome) (fig. 2). The results of enrichment analyses suggested the potential impact of cilium organization and centrosome amplification on the evolutionary development of kidneys in DMK mammals, given that the primary cilium is an organelle that plays a key role in cell signaling and is mainly associated with SHH signaling, which has been proposed to be involved in duplex kidney formation (San Agustin et al. 2016; Elliott and Brugmann 2019), and amplification of the centrosome could disrupt renal development (Dionne et al. 2018). The etiology of most duplex kidneys can be traced back to ureteral induction, although both the ureter and kidney belong to the urogenital system (Uetani et al. 2009). Thus, the evolutionary development of the DMK is thought to benefit from the evolution of the entire urogenital system. Additionally, two genes (*FAT4* and *TBC1D32*) were found evolved rapidly either in DMK mammals or in non-DMK mammals, *FAT4* is a key gene in vertebrate planar cell polarity and its loss can overactivate GDNF-RET signaling, causing premature branching with incomplete duplication (Zhang et al. 2019). *TBC1D32* was found to account for the duplex kidney formation as a part of a ciliopathy phenotype (San Agustin et al. 2016). Two convergent amino acid substitutions, which were in crucial functional domains of protein TBC1D32, were detected among species with DMKs. And the detail of amino acid changes is discussed later. This result suggests the same gene may underlie the evolution of both species with DMKs and with other structural types of kidneys for resisting to stress (fig. 2*a*) (Valenzano et al. 2015).

Second, comparative analysis of the DMK and non-DMK species revealed that six genes are under positive selection in DMK mammals (table 3). Among them, six positively selected sites in four genes were found in key domains of corresponding proteins. For example, the ARM (Armadillo repeat) domain is a repetitive sequence about 40 residues long, multiple copies of which form an alpha solenoid structure; the domain can control act branching and bundling and is pivotal for transducing WNT signals during embryonic development (protein CTNNB1, site 167) (Nusse and Clevers 2017). In addition, the LRRNT (leucine-rich repeat N-terminal) domain is rich in the hydrophobic amino acid leucine and related to the formation of protein–protein interactions (protein SLIT2, site 279) (Kobe and Kajava 2001). The EGF (epidermal growth factor) domain occurs in many tandem copies in protein and fold together into a functional unit (protein SLIT2, site 984) (Rao et al. 1995). The Fn3 (fibronectin type III) domain consists of many proteins related to ligand binding (protein ROBO2, site 597) (Koide et al. 1998); the B9-C2 (ciliary basal body–associated, B9 protein) domain exists in proteins involved in the ciliary basal body (protein MKS1, site 261 and 364) (Dawe et al. 2007). In addition, 10 out of 15 positively selected sites with radical amino acid property changes were shown to impact the structure and function of their corresponding proteins. These positively selected sites in the crucial domains of proteins may be evidence for the molecular convergence of mammals with DMKs. Furthermore, the enrichment suggested that the biological processes involving epithelial tube morphogenesis, the regulation of neurogenesis, and axon guidance—all of which positively selected genes are enriched in—may be closely related to the adaptive evolution of DMKs in mammals. Among them, Roundabout Guidance Receptor 2 (*ROBO2*) plays a role in axon guidance and cell migration as a transmembrane receptor for Slit Guidance Ligand 2 (SLIT2). SLIT proteins function in axon guidance and neuronal migration. Knock out of *ROBO2* or *SLIT2* in mice can lead to an abnormal GDNF expression domain, which causes duplex kidney formation (Grieshammer et al. 2004). MKS Transition Zone Complex Subunit 1 (*MKS1*) is necessary for ciliated epithelial cells to form primary cilia, and *MKS1* mutant can also form the duplex kidney, which is a part of a ciliopathy phenotype (San Agustin et al. 2016). Inactivation of Catenin Beta 1 (*CTNNB1*) in the nephric duct or hypoxia-induced reduction of it can result in duplex kidneys (Bridgewater et al. 2008; Wilkinson et al. 2015).

Finally, in this study, 6 out of 12 unique convergent amino acid changes were identified in key domains of their respective proteins (fig. 3). Of them, the dynein heavy chain region D6 P-loop domain comes from a chain involved in ATPase activity, microtubule binding ability, cilia and flagella movement (Mocz and Gibbons 2001), and the ROMI (broad-minded) domain's interaction with cell cycle–related kinase (CCRK) proteins, which together regulate ciliary membrane and axonal growth (Ko et al. 2010); the AAA+ lid domain represents the C terminus of AAA domains from dynein heavy chain D3 (Mocz and Gibbons 2001). Moreover, two unique convergent amino acid changes (e.g., D3991G in protein DNAH5 and A38T in protein SLIT2) were inferred to be deleterious or damaging to protein properties and potentially affect their functions. DYNEIN, AXONEMAL, HEAVY CHAIN 5 (*DNAH5*) encodes a kinesin protein, and mutation in it can cause primary ciliary dyskinesia type 3 (Olbrich et al. 2002). Mutants in mice lacking *SLIT2* or *ROBO2* form additional ureteral buds, which can lead to phenotypes such as duplex kidney (Grieshammer et al. 2004). Taken together, these DMK species-specific convergent amino acid substitutions in genes associated with duplex kidney formation may account for the phenotypical convergence among them.

However, renal structural development is an intricate process; future studies based on multiomics analysis are needed to shed light on the deeper and more comprehensive molecular mechanisms underlying the evolution of DMKs in mammals. Experimental verification is also essential, if conditions permit.

## Conclusion

With regard to the origin and macroevolution of renal structures across mammals, we found that the UK was the ancestral state in mammals, and that multilobar kidneys independently evolved multiple times. In addition, mammals with larger body sizes or living in aquatic environments tended to evolve DMKs. With regard to molecular evolution, comparative genomic analyses of 45 genes related to duplex/multiplex kidney formation in 41 mammals revealed that the convergent evolution of mammals with DMKs may be resulted from the REGs associated with cilium assembly and centrosome amplification, the positively selected genes involved in epithelial tube morphogenesis, regulation of neurogenesis and axon guidance, and six key convergent amino acid substitutions in functional domains of proteins. Taken together, our study provides novel insights into the origin and evolution of renal structures, especially DMKs, and offers a better understanding of the pathology of duplex/multiplex kidneys in human from macroecological and micromolecular perspectives.

## Materials and Methods

### Species Coverage and Sequence Acquisition

The ancestral state reconstruction and correlation analyses covered a total of 62 mammals from 13 orders: Cetartiodactyla (*Balaenoptera acutorostrata*, *Balaenoptera musculus*, *Tursiops truncatus*, *Orcinus orca*, *Delphinapterus leucas*, *Physeter catodon*, *Monodon monoceros*, *Globicephala melas*, *Phocoena sinus*, *Lagenorhynchus obliquidens*, *Lipotes vexillifer*, *Neophocaena phocaenoides*, *Bos taurus*, *Bos indicus*, *Bison bison*, *Bubalus bubalis*, *Ovis aries*, *Hippopotamus amphibius*, *Camelus dromedarius*, *Camelus bactrianus*, *Capra hircus*, and *Sus scrofa*), Carnivora (*Leptonychotes weddellii*, *Neomonachus schauinslandi*, *Mirounga leonina*, *Eumetopias jubatus*, *Zalophus californianus*, *Odobenus rosmarus*, *Callorhinus ursinus*, *Lutra lutra*, *Enhydra lutris*, *Ursus maritimus*, *Ursus americanus*, *Ursus arctos*, *Felis catus*, *Canis lupus familiaris*, *Panthera pardus*, *Panthera tigris*, and *Phoca vitulina*), Perissodactyla (*Diceros bicornis*, *Ceratotherium simum*, *Equus caballus*, and *Equus asinus*), Chiroptera (*Rhinolophus ferrumequinum*), Eulipotyphla (*Sorex araneus*, *Erinaceus europaeus*), Rodentia (*Castor canadensis*, *Mus musculus*, *Cavia porcellus*, and *Heterocephalus glaber*), Primates (*Homo sapiens*, *Macaca mulatta*, *Aotus nancymaae*, and *Papio anubis*), Proboscidea (*Loxodonta africana*, *Elephas maximus*), Sirenia (*Trichechus manatus*), Macroscelidea (*Elephantulus edwardii*), Cingulata (*Dasypus novemcinctus*), Marsupialia (*Phascolarctos cinereus*, *Monodelphis domestica*), and Monotremata (*Ornithorhynchus anatinus*) (supplementary table S1, Supplementary Material online). Three categories of renal structural types were classified: UK, CMK, and DMK. Among them, 41 species covering 13 orders were used in the subsequent evolutionary analyses (supplementary fig. S2, Supplementary Material online).

The gene set related to duplex/multiplex kidney formation in humans was collected from a review article published in 2020 (Kozlov and Schedl 2020) and a database using the keyword “duplex kidney” (http://www.informatics.jax.org/mp/annotations/MP:0004017) (supplementary table S9, Supplementary Material online). The protein-coding sequences (CDS) were then downloaded from the NCBI database (https://www.ncbi.nlm.nih.gov/). In addition, the partial or unannotated CDS was further verified using BlastN searches with custom perl scripts. The longest transcript was retained for each gene in this analysis. Finally, 45 one-to-one orthologous genes among 41 species were used for the analysis described below. To obtain better quality sequence alignments, we performed multiple sequence alignments for each orthologous gene using PRANK v.170427 (Löytynoja 2014) combined with MACSE v2 (Ranwez et al. 2018) in the codon mode. The aligned sequences were then trimmed using Gblocks v0.91 (Talavera and Castresana 2007) with default settings.

### Ancestral State Reconstruction

To reconstruct the ancestral state of mammals, we took the phenotype data from a 1996 research article (Beuchat 1996) and downloaded the corresponding phylogenetic tree file from TimeTree (http://www.timetree.org/) (Kumar et al. 2017) (supplementary fig. S1, Supplementary Material online). Then, we carried out an ancestral state reconstruction using Bayesian Binary MCMC (BBM) analysis implemented in RASP v4.2 (Yu et al. 2020). The program calculated several probable results using corresponding models (e.g., DEC, DEC + J, DIVALIKE, DIVALIKE + J, BAYAREALIKE, and BAYAREALIKE + J), and we chose the most reliable one, given that the reconstruction with chosen model possessed the lowest AIC value and the highest AICc_wt value (i.e., the fixed model DEC + J).

### Association Analysis of Life History Traits and Renal Structural Phenotypes

We collected the life history traits from different reliable resources, such as the body mass of 62 mammals from the PanTHERIA database (Jones et al. 2009) and feeding and habitat data from the ADW database (https://animaldiversity.org/).

We used several approaches in this analysis. Regarding the relationship between body mass and renal structures in mammals, PGLS regression was employed in the R package Caper (Orme et al. 2013). We transferred the renal structural phenotype into a binary state: discrete and non-DMKs. In terms of discrete variables (habitats and diets), we conducted the function phyloglm in the R package phylolm version 2.6 with the IG10 phylogenetic generalized linear model (Tung Ho and Ané 2014). To further explore the independent and dependent evolution between habitats and renal structures in mammals, we implemented the analysis using the R package phytools with maximum likelihood models to test how one trait affects the transition rates of another trait (Singh et al. 2012).

### Molecular Evolution Analyses

To test the selective pressure on genes, we estimated the ratio of nonsynonymous (*d*_N_)/synonymous (*d*_S_) substitution rates (*d*_N_/*d*_S_) implemented in the CodeML program of the PAML software package v4.9 (Yang 2007). The ratio is also called *ω* value, and *ω* < 1, *ω* = 1, and *ω* > 1 mean purified selection, neutral evolution, and positive selection, respectively. The phylogenetic tree used here was downloaded from TimeTree (supplementary fig. S2, Supplementary Material online). The ancestral and terminal branches of all species with DMKs were labelled as foreground branches, other branches as background branches.

We detected the rapid evolution of genes using the branch model in 41 mammals. The one-ratio model assumes that all branches on the phylogenetic tree have the same *ω* ratio against an alternative hypothesis (two-ratio model), which allows the *ω* ratio of the foreground branch to differ from that of the background branch. Then, we executed a likelihood ratio test (LRT) with a chi-square distribution and applied the FDR correction for multiple testing.

The branch–site model was then used to detect the positively selected genes and amino acids in mammals. All positively selected sites in this analysis were identified using a Bayes Empirical Bayes (BEB) analysis with posterior probabilities ≥ 0.80 (Yang et al. 2005). Furthermore, we estimated the amino acid physicochemical properties of crucial sites using TreeSAAP software, and sites with values of 6–8 were considered to have radical amino acid property changes (Woolley et al. 2003).

### Convergent Amino Acid Substitution Detection

Convergent phenotypic characteristics can result from specific substitutions that independently evolved in different species (Natarajan et al. 2015). We detected the molecular basis of convergent evolution in species with DMK by identifying the unique convergent amino acid substitutions based on sequence alignments using FasParser2 with the “Segregate” function (Sun 2018). If we identified the same amino acid changes in at least two lineages with DMKs, then these amino acid changes were considered to be group-specific convergent amino acid changes.

### Functional Domain Searching and Key Site Labeling on 3D Structures of Proteins

To verify whether the positively selected sites or unique convergent amino acid sites were located in the key domains of proteins, we searched the UniProt database (https://www.uniprot.org/) (UniProt Consortium 2019) and mapped the sites onto the 3D protein structures predicted from SWISS-MODEL (https://swissmodel.expasy.org/) by EzMol (http://www.sbg.bio.ic.ac.uk/ezmol/) (Reynolds et al. 2018; Waterhouse et al. 2018).

### Gene Functions and Signaling Pathway Annotation and Enrichment

The functional enrichment analyses in GO for biological process (BP), cellular component (CC), molecular function (MF), and Kyoto Encyclopedia of Genes and Genomes (KEGG) were performed in R package clusterProfiler (Yu et al. 2012). FDR was performed using the Benjamini and Hochberg (BH) method (Benjamini and Hochberg 1995).

### Functional Effects Estimating of Convergent Amino Acid Substitutions

We tested the potential effects of these convergent amino acid substitutions on the corresponding proteins using PolyPhen-2 (Adzhubei et al. 2010) and PROVEAN (Choi and Chan 2015). The output of PolyPhen-2 is classified as benign, possibly damaging (low confidence), and probably damaging (high confidence), along with score ranging from 0 (benign) to 1 (damaging). In order to obtain high balanced accuracy, the cut-off value of the PROVEAN score is set to −2.5. The protein variant is predicted to be deleterious when the score is less than or equal to the predefined threshold.

## Supplementary Material

evad075_Supplementary_Data

## Data Availability

All data underlying this article are available from this article and its online Supplementary material.

## References

[evad075-B1] Adzhubei IA , et al 2010. A method and server for predicting damaging missense mutations. Nat Methods. 7:248–249. 20354512 10.1038/nmeth0410-248 PMC2855889

[evad075-B2] Benjamini Y , Hochberg Y. 1995. Controlling the false discovery rate: a practical and powerful approach to multiple testing. J R Stat Soc Series B Stat Methodol. 57:289–300.

[evad075-B3] Bennett AF . 1997. Adaptation and evolution of physiological characters. In: Dantzler WH, editor. Handbook of physiology, comparative physiology. New York: Oxford University Press. p. 1–16.

[evad075-B4] Berglund LG , et al 2020. Physiological and toxicological considerations. In: Industrial ventilation design guidebook. London: Academic Press. p. 111–226.

[evad075-B5] Beuchat CA . 1996. Structure and concentrating ability of the mammalian kidney: correlations with habitat. Am J Physiol. 271:R157–R179. 8760217 10.1152/ajpregu.1996.271.1.R157

[evad075-B6] Beuchat C . 2002. Kidney, structure and function. In: Encyclopedia of marine mammals. San Diego: Academic Press. p. 646–649.

[evad075-B7] Bokma F . 2008. Detection of “punctuated equilibrium” by Bayesian estimation of speciation and extinction rates, ancestral character states, and rates of anagenetic and cladogenetic evolution on a molecular phylogeny. Evolution 62:2718–2726. 18752617 10.1111/j.1558-5646.2008.00492.x

[evad075-B8] Bridgewater D , et al 2008. Canonical WNT/β-catenin signaling is required for ureteric branching. Dev Biol. 317:83–94. 18358465 10.1016/j.ydbio.2008.02.010

[evad075-B9] Calder WA 3rd , Braun EJ. 1983. Scaling of osmotic regulation in mammals and birds. Am J Physiol. 244:R601–R606. 6846567 10.1152/ajpregu.1983.244.5.R601

[evad075-B10] Choi Y , Chan AP. 2015. PROVEAN Web server: a tool to predict the functional effect of amino acid substitutions and indels. Bioinformatics 31:2745–2747. 25851949 10.1093/bioinformatics/btv195 PMC4528627

[evad075-B11] Dantzler WH . 1989. Comparative physiology of the vertebrate kidney. Berlin: Springer-Verlag.

[evad075-B12] Dantzler WH , Braun EJ. 1980. Comparative nephron function in reptiles, birds, and mammals. Am J Physiol. 239:R197–R213. 7001920 10.1152/ajpregu.1980.239.3.R197

[evad075-B13] Dawe HR , et al 2007. The Meckel–Gruber syndrome proteins MKS1 and meckelin interact and are required for primary cilium formation. Hum Mol Genet. 16:173–186. 17185389 10.1093/hmg/ddl459

[evad075-B14] Dionne LK , et al 2018. Centrosome amplification disrupts renal development and causes cystogenesis. J Cell Biol. 217:2485–2501. 29895697 10.1083/jcb.201710019 PMC6028550

[evad075-B15] Domning DP . 2001. The earliest known fully quadrupedal sirenian. Nature 413:625–627. 11675784 10.1038/35098072

[evad075-B16] Dwyer TM , Schmidt-Nielsen B. 2003. The renal pelvis: machinery that concentrates urine in the papilla. News Physiol Sci. 18:1–6. 12531923 10.1152/nips.1416.2002

[evad075-B17] Elliott KH , Brugmann SA. 2019. Sending mixed signals: cilia-dependent signaling during development and disease. Dev Biol. 447:28–41. 29548942 10.1016/j.ydbio.2018.03.007 PMC6136992

[evad075-B18] Finco DR . 1997. Kidney function. In: Clinical biochemistry of domestic animals. San Diego: Academic Press. p. 441–484.

[evad075-B19] Fisher RA . 1958. The genetical theory of natural selection. New York: Dover Publications.

[evad075-B20] Foote AD , et al 2015. Convergent evolution of the genomes of marine mammals. Nat Genet. 47:272–275. 25621460 10.1038/ng.3198 PMC4644735

[evad075-B21] Gaeth A , Short R, Renfree M. 1999. The developing renal, reproductive, and respiratory systems of the African elephant suggest an aquatic ancestry. Proc Natl Acad Sci U S A. 96:5555–5558. 10318922 10.1073/pnas.96.10.5555 PMC21898

[evad075-B22] Grieshammer U , et al 2004. SLIT2-mediated ROBO2 signaling restricts kidney induction to a single site. Dev Cell. 6:709–717. 15130495 10.1016/s1534-5807(04)00108-x

[evad075-B23] Hao Y , Qu Y, Song G, Lei F. 2019. Genomic insights into the adaptive convergent evolution. Curr Genomics. 20:81–89. 31555059 10.2174/1389202920666190313162702 PMC6728901

[evad075-B24] Hodson J . 1972. The lobar structure of the kidney. Br J Urol. 44:246–261. 5041555 10.1111/j.1464-410x.1972.tb10072.x

[evad075-B25] Jamison RL , Kriz W. 1982. Urinary concentrating mechanism: structure and function. New York: Oxford University Press.

[evad075-B26] Jones KE , et al 2009. PanTHERIA: a species-level database of life history, ecology, and geography of extant and recently extinct mammals: Ecological Archives E090-184. Ecology 90:2648–2648.

[evad075-B27] King JE . 1991. Seals of the world. New York: Cornell University Press.

[evad075-B28] Ko HW , et al 2010. Broad-minded links cell cycle-related kinase to cilia assembly and hedgehog signal transduction. Dev Cell. 18:237–247. 20159594 10.1016/j.devcel.2009.12.014 PMC2830714

[evad075-B29] Kobe B , Kajava AV. 2001. The leucine-rich repeat as a protein recognition motif. Curr Opin Struc Biol. 11:725–732. 10.1016/s0959-440x(01)00266-4 11751054

[evad075-B30] Koide A , Bailey CW, Huang X, Koide S. 1998. The fibronectin type III domain as a scaffold for novel binding proteins. J Mol Biol. 284:1141–1151. 9837732 10.1006/jmbi.1998.2238

[evad075-B31] Kozlov VM , Schedl A. 2020. Duplex kidney formation: developmental mechanisms and genetic predisposition. F1000Res 9:F1000 Faculty Rev-2. 10.12688/f1000research.19826.1 PMC6945105 32030122

[evad075-B32] Kriz W . 1981. Structural organization of the renal medulla: comparative and functional aspects. Am J Physiol. 241:R3–R16. 7018270 10.1152/ajpregu.1981.241.1.R3

[evad075-B33] Kriz W , Bankir L. 1988. A standard nomenclature for structures of the kidney. The renal commission of the international union of physiological sciences (IUPS). Kidney Int. 33:1–7. 3352156 10.1038/ki.1988.1

[evad075-B34] Kriz W , Kaissling B, Sa G. 2008. Structural and functional organization of the kidney. In: The kidney: physiology and pathophysiology. London: Academic Press. p. 479–563.

[evad075-B35] Kumar S , Stecher G, Suleski M, Hedges SB. 2017. TimeTree: a resource for timelines, timetrees, and divergence times. Mol Biol Evol. 34:1812–1819. 28387841 10.1093/molbev/msx116

[evad075-B36] Lavergne A , Douzery E, Stichler T, Catzeflis FM, Springer MS. 1996. Interordinal mammalian relationships: evidence for paenungulate monophyly is provided by complete mitochondrial 12S rRNA sequences. Mol Phylogenet Evol. 6:245–258. 8899726 10.1006/mpev.1996.0074

[evad075-B37] Löytynoja A . 2014. Phylogeny-aware alignment with PRANK. In: Multiple sequence alignment methods. Totowa: Humana Press. p. 155–170. 10.1007/978-1-62703-646-7_10 24170401

[evad075-B38] Mahasen LMA . 2016. Evolution of the kidney. Anat Physiol Biochem. 1:1–6.

[evad075-B39] Marieb EN , Hoehn K. 2006. Urinary system. In: Essentials of human anatomy and physiology. San Francisco: Pearson Benamin Cummings Publishing. p. 501–526.

[evad075-B40] Marshall E Jr , Smith HW. 1930. The glomerular development of the vertebrate kidney in relation to habitat. Biol Bull. 59:135–153.

[evad075-B41] Matzke NJ . 2014. Model selection in historical biogeography reveals that founder-event speciation is a crucial process in island clades. Syst Biol. 63:951–970. 25123369 10.1093/sysbio/syu056

[evad075-B42] Mocz G , Gibbons I. 2001. Model for the motor component of dynein heavy chain based on homology to the AAA family of oligomeric ATPases. Structure 9:93–103. 11250194 10.1016/s0969-2126(00)00557-8

[evad075-B43] Natarajan C , et al 2015. Convergent evolution of hemoglobin function in high-altitude Andean waterfowl involves limited parallelism at the molecular sequence level. PLoS Genet. 11:e1005681. 26637114 10.1371/journal.pgen.1005681 PMC4670201

[evad075-B44] Nusse R , Clevers H. 2017. Wnt/β-catenin signaling, disease, and emerging therapeutic modalities. Cell 169:985–999. 28575679 10.1016/j.cell.2017.05.016

[evad075-B45] Olbrich H , et al 2002. Mutations in *DNAH5* cause primary ciliary dyskinesia and randomization of left-right asymmetry. Nat Genet. 30:143–144. 11788826 10.1038/ng817

[evad075-B46] Oliver J . 1968. Nephrons and kidneys: a quantitative study of developmental and evolutionary mammalian renal architectonics. New York: Hoeber Medical Division Harper & Row.

[evad075-B47] Orme D , et al 2013. The caper package: comparative analysis of phylogenetics and evolution in R. http://cran.r-project.org/package=caper.

[evad075-B48] Ortiz RM . 2001. Osmoregulation in marine mammals. J Exp Biol. 204:1831–1844. 11441026 10.1242/jeb.204.11.1831

[evad075-B49] Pascoal S , et al 2014. Rapid convergent evolution in wild crickets. Curr Biol. 24:1369–1374. 24881880 10.1016/j.cub.2014.04.053

[evad075-B50] Pfeiffer CJ . 1997. Renal cellular and tissue specializations in the bottlenose dolphin (*Tursiops truncatus*) and beluga whale (*Delphinapterus leucas*). Aquat Mamm. 23:75–84.

[evad075-B51] Prothero J . 1984. Organ scaling in mammals: the kidneys. Comp Biochem Phys A Comp Physiol. 77:133–138. 10.1016/0300-9629(84)90024-0 6141021

[evad075-B52] Ranwez V , Douzery EJ, Cambon C, Chantret N, Delsuc F. 2018. MACSE V2: toolkit for the alignment of coding sequences accounting for frameshifts and stop codons. Mol Biol Evol. 35:2582–2584. 30165589 10.1093/molbev/msy159 PMC6188553

[evad075-B53] Rao Z , et al 1995. The structure of a Ca2^+^-binding epidermal growth factor-like domain: its role in protein-protein interactions. Cell 82:131–141. 7606779 10.1016/0092-8674(95)90059-4

[evad075-B54] Reynolds CR , Islam SA, Sternberg MJ. 2018. Ezmol: a web server wizard for the rapid visualization and image production of protein and nucleic acid structures. J Mol Biol. 430:2244–2248. 29391170 10.1016/j.jmb.2018.01.013 PMC5961936

[evad075-B55] Rouiller C . 1969. General anatomy and histology of the kidney. In: The kidney. New York: Academic Press. p. 61–156.

[evad075-B56] San Agustin JT , et al 2016. Genetic link between renal birth defects and congenital heart disease. Nat Commun. 7:11103. 27002738 10.1038/ncomms11103 PMC4804176

[evad075-B57] Scheiner SM . 1992. The evolution of life histories. Science 258:1820–1821. 17831664 10.1126/science.258.5089.1820

[evad075-B58] Schmidt–Nielsen B . 1987. The renal pelvis. Kidney Int. 31:621–628. 3550232 10.1038/ki.1987.43

[evad075-B59] Singh RS , Xu J, Kulathinal RJ. 2012. Rapidly evolving genes and genetic systems. Oxford: Oxford University Press.

[evad075-B60] Straus W , Arcadia J. 1958. Urinary system. In: Primatologia. New York: Karger. p. 507–541.

[evad075-B61] Sun Y-B . 2018. Fasparser2: a graphical platform for batch manipulation of tremendous amount of sequence data. Bioinformatics 34:2493–2495. 29514176 10.1093/bioinformatics/bty126

[evad075-B62] Talavera G , Castresana J. 2007. Improvement of phylogenies after removing divergent and ambiguously aligned blocks from protein sequence alignments. Syst Biol. 56:564–577. 17654362 10.1080/10635150701472164

[evad075-B63] Tung Ho Ls , Ané C. 2014. A linear-time algorithm for Gaussian and non-Gaussian trait evolution models. Syst Biol. 63:397–408. 24500037 10.1093/sysbio/syu005

[evad075-B64] Uetani N , et al 2009. Maturation of ureter-bladder connection in mice is controlled by LAR family receptor protein tyrosine phosphatases. J Clin Invest. 119:924–935. 19273906 10.1172/JCI37196 PMC2662538

[evad075-B65] UniProt Consortium . 2019. UniProt: a worldwide hub of protein knowledge. Nucleic Acids Res. 47:D506–D515. 30395287 10.1093/nar/gky1049 PMC6323992

[evad075-B66] Valenzano DR , et al 2015. The African turquoise killifish genome provides insights into evolution and genetic architecture of lifespan. Cell 163:1539–1554. 26638078 10.1016/j.cell.2015.11.008 PMC4684691

[evad075-B67] Verhaegen M . 1993. Aquatic versus Savanna: comparative and paled-environmental evidence. Nutr Health. 9:165–191. 8183486 10.1177/026010609300900304

[evad075-B68] Waterhouse A , et al 2018. SWISS-MODEL: homology modelling of protein structures and complexes. Nucleic Acids Res. 46:W296–W303. 29788355 10.1093/nar/gky427 PMC6030848

[evad075-B69] Wilkinson LJ , et al 2015. Renal developmental defects resulting from in utero hypoxia are associated with suppression of ureteric β-catenin signaling. Kidney Int. 87:975–983. 25587709 10.1038/ki.2014.394

[evad075-B70] Williams MF . 1997. The adaptive significance of endothermy and salt excretion amongst the earliest archosaurs. Speculations Sci Technol. 20:237–247.

[evad075-B71] Williams MF . 2006. Morphological evidence of marine adaptations in human kidneys. Med Hypotheses. 66:247–257. 16263222 10.1016/j.mehy.2005.09.024

[evad075-B72] Woolley S , Johnson J, Smith MJ, Crandall KA, McClellan DA. 2003. TreeSAAP: selection on amino acid properties using phylogenetic trees. Bioinformatics 19:671–672. 12651734 10.1093/bioinformatics/btg043

[evad075-B73] Wursig B , Perrin WF. 2009. Encyclopedia of marine mammals. San Diego: Academic Press.

[evad075-B74] Yang Z . 2007. PAML 4: phylogenetic analysis by maximum likelihood. Mol Biol Evol. 24:1586–1591. 17483113 10.1093/molbev/msm088

[evad075-B75] Yang Z , Wong WS, Nielsen R. 2005. Bayes Empirical Bayes inference of amino acid sites under positive selection. Mol Biol Evol. 22:1107–1118. 15689528 10.1093/molbev/msi097

[evad075-B76] Yu Y , Blair C, He X. 2020. RASP 4: ancestral state reconstruction tool for multiple genes and characters. Mol Biol Evol. 37:604–606. 31670774 10.1093/molbev/msz257

[evad075-B77] Yu G , Wang L-G, Han Y, He Q-Y. 2012. Clusterprofiler: an R package for comparing biological themes among gene clusters. OMICS 16:284–287. 22455463 10.1089/omi.2011.0118 PMC3339379

[evad075-B78] Zhang H , et al 2019. FAT4 fine-tunes kidney development by regulating RET signaling. Dev Cell. 48:780–792. 30853441 10.1016/j.devcel.2019.02.004 PMC6766079

