# Supplementary material for: The Evolution of the Discrete Multirenculate Kidney in Mammals from Ecological and Molecular Perspectives
Source: Genome Biol Evol. 2023 May 9;15(5):evad075. doi: 10.1093/gbe/evad075 (PMC10198776; doi:10.1093/gbe/evad075)
Supplement: evad075_Supplementary_Data [file evad075_supplementary_data.docx]

**Supplementary Information**

**Supplementary Figures**

**
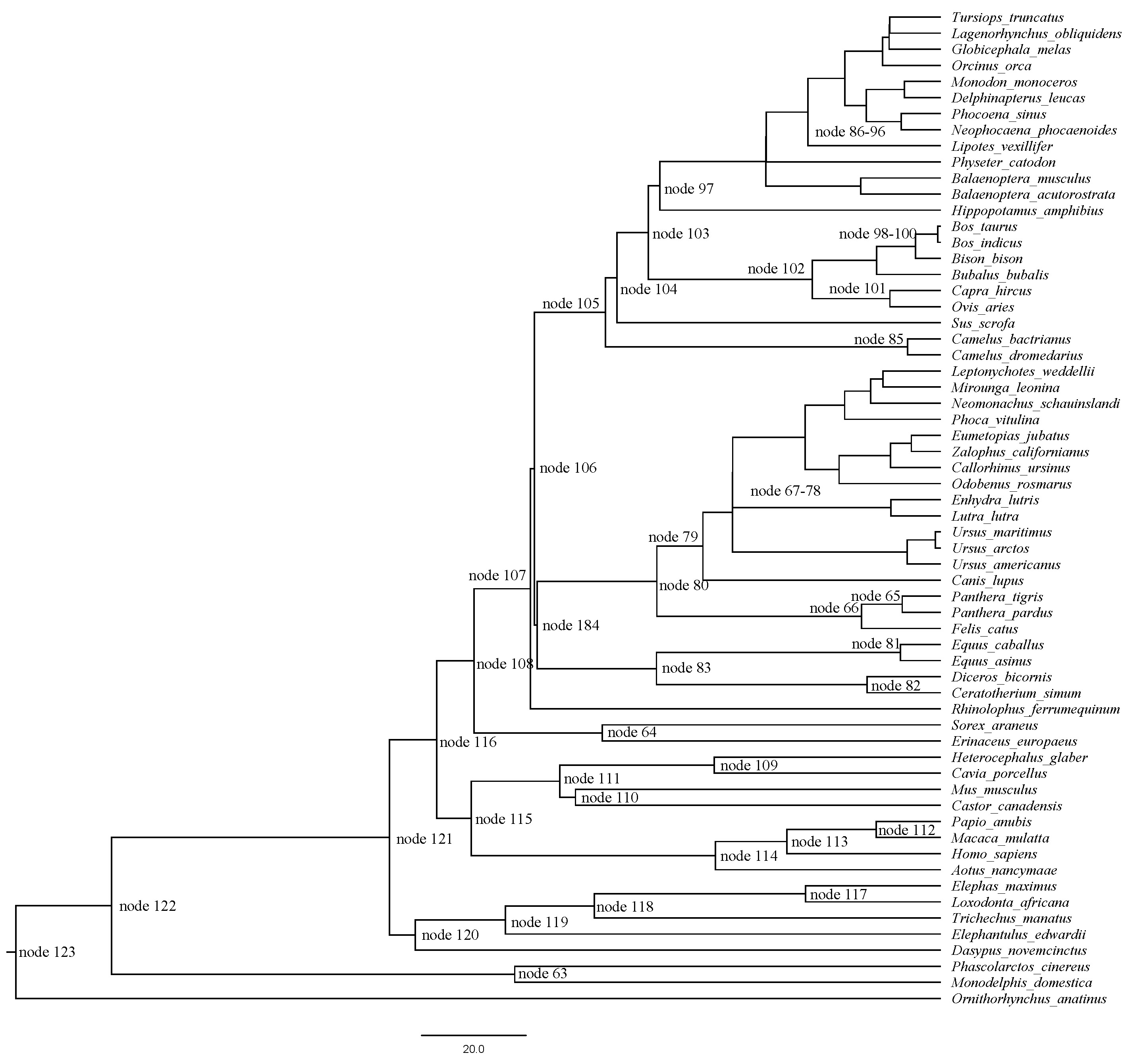
**

**Fig S1** The phylogenetic tree with labeled node about 62 mammals from TimeTree (http://www.timetree.org/) used in ancestral state reconstruction and correlation analysis.


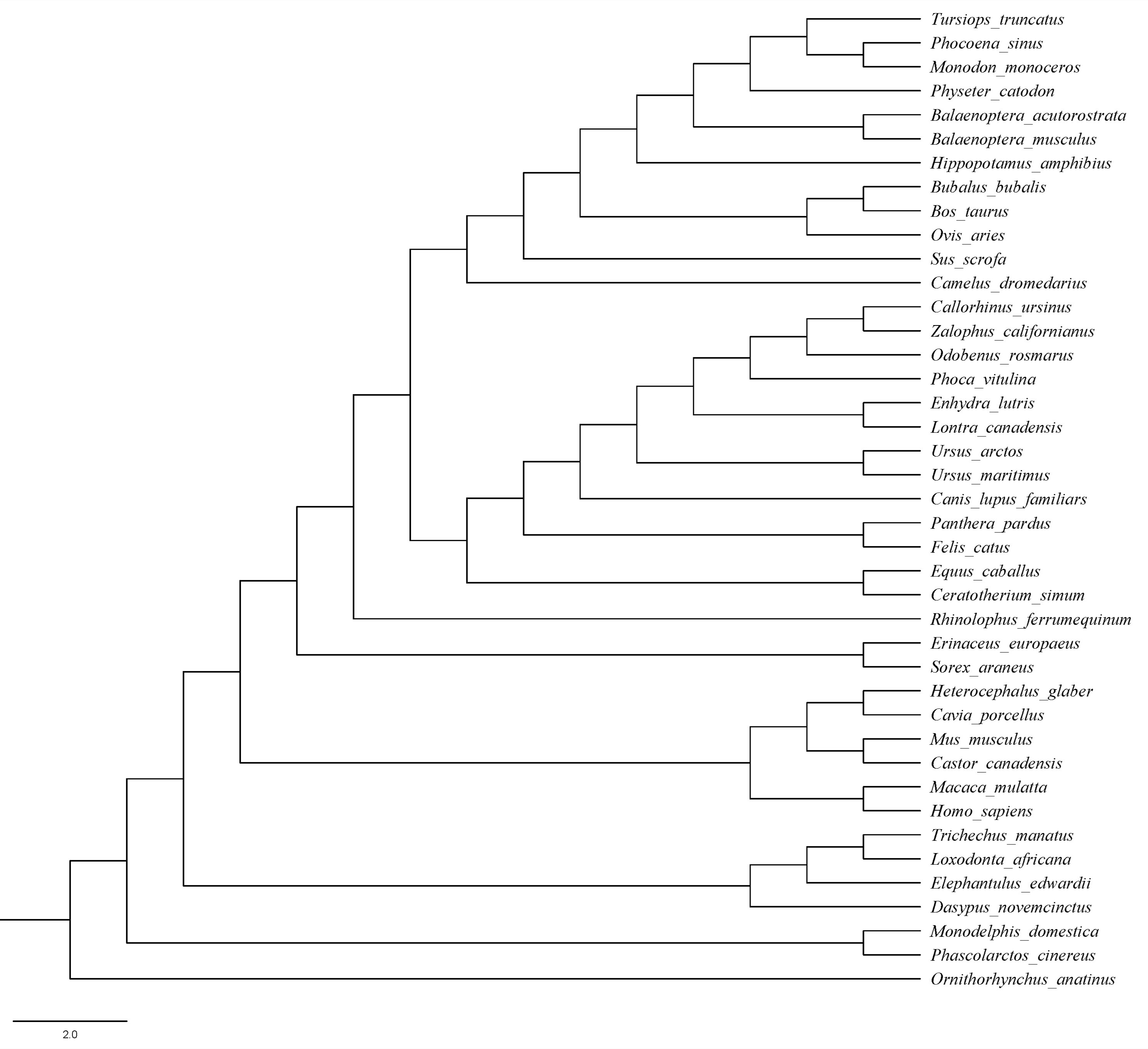


**Fig S2** The phylogenetic tree about 41 mammals used in molecular evolutionary analyses.


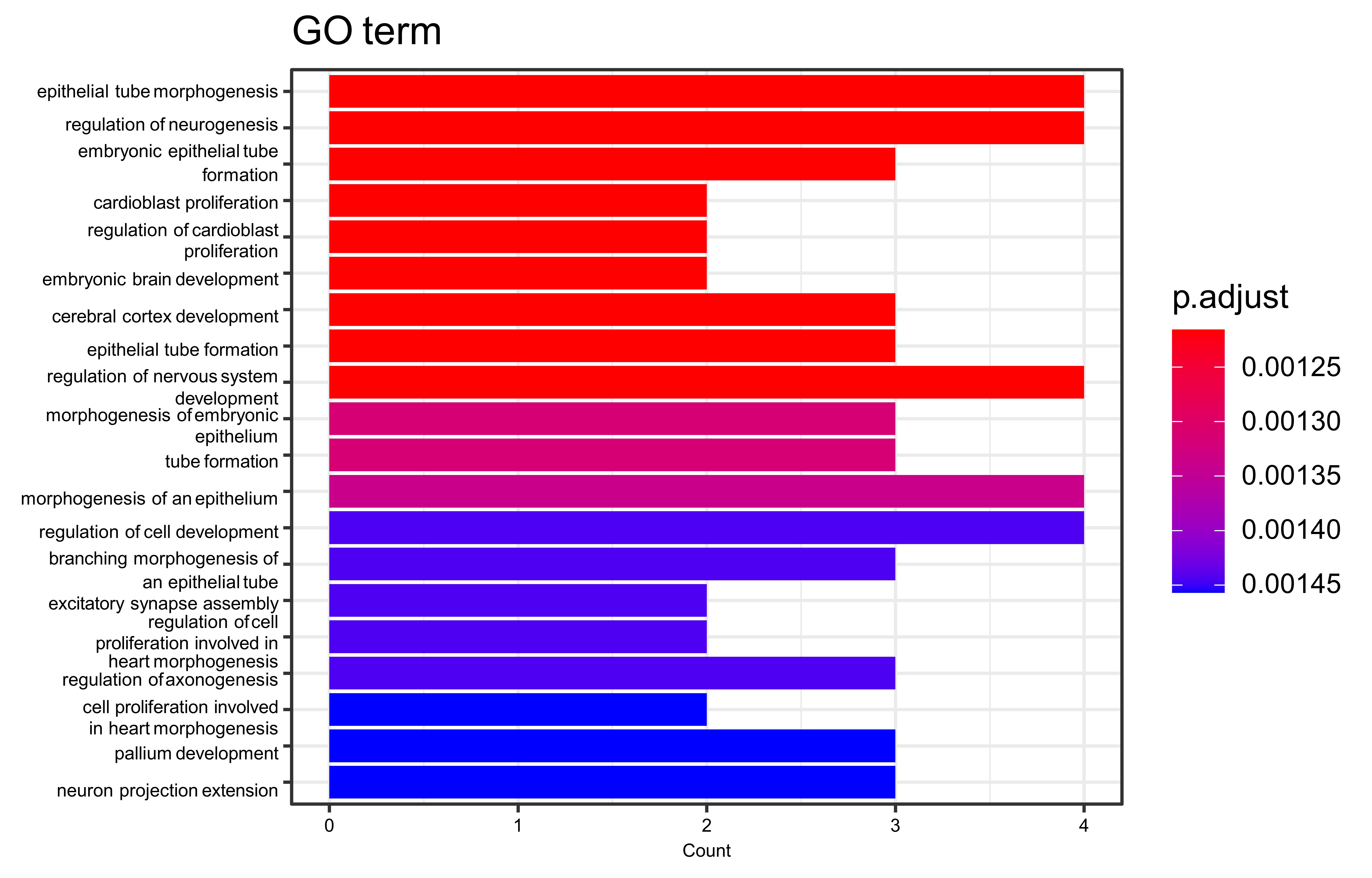


**Fig S3** GO enrichment (Biological Process, BP) plot of positively selected genes in DMK mammals.


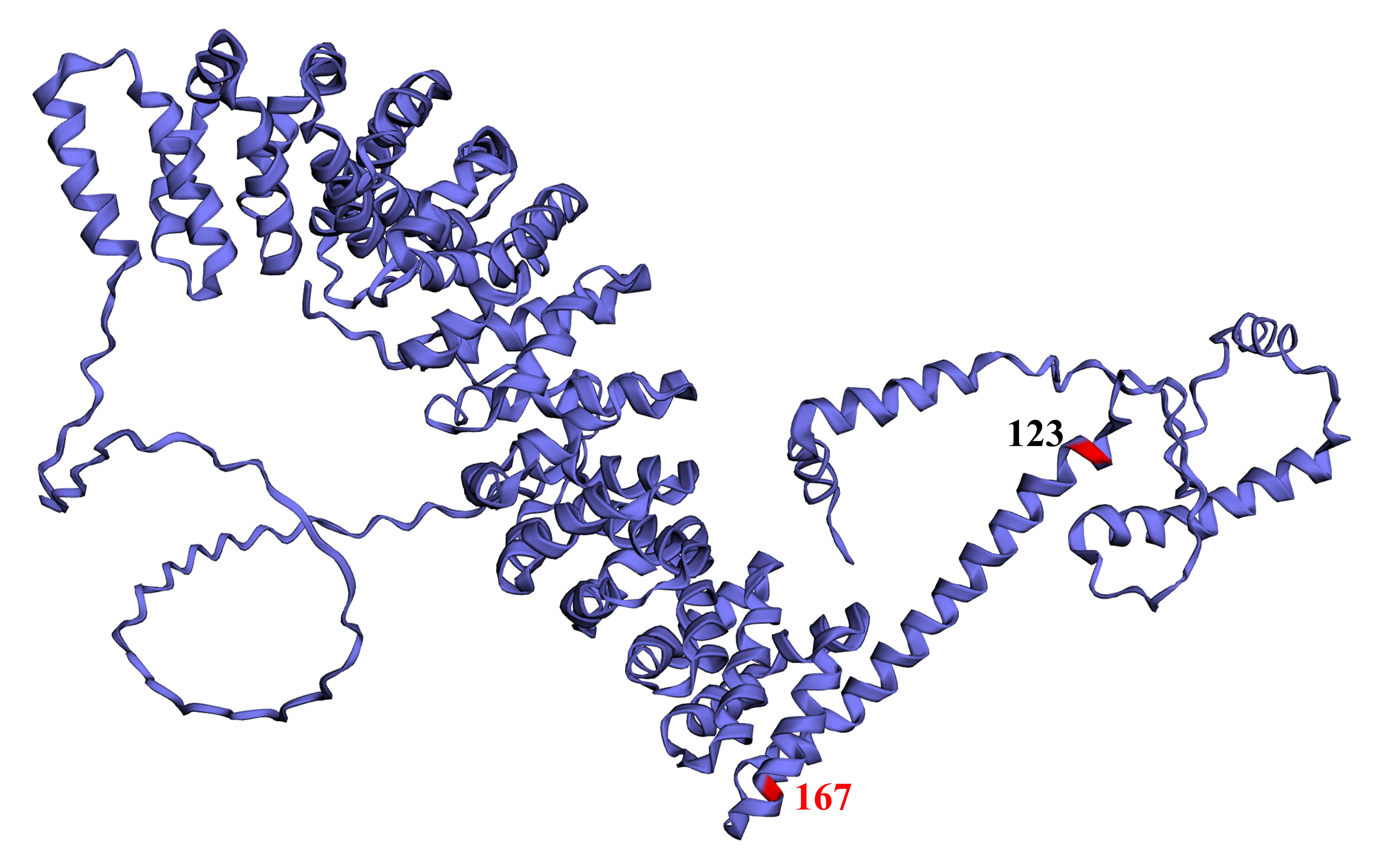


**CTNNB1**


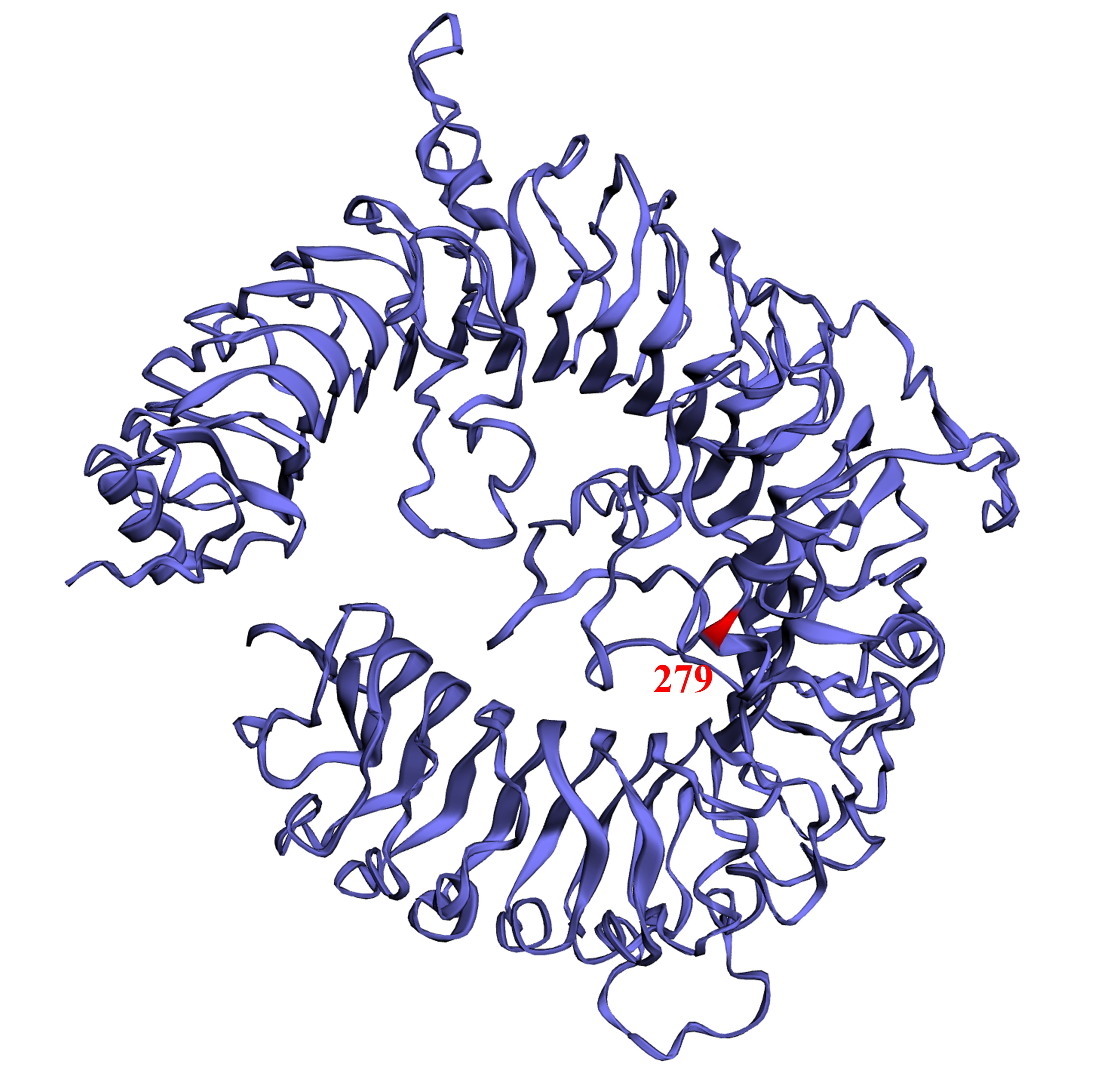


**SLIT2**

**Fig S4** Distribution of positive selection sites on the 3D structure of the partial proteins (protein CTNNB1 and protein SLIT2)

**Supplementary Tables**

**Table S1 Datasets of 62 mammals in present study.**

| **Order** | **Species** | **Renal structures** | **Lg (Body mass)** | **Diet** | **Habitats** |
| --- | --- | --- | --- | --- | --- |
| Cetartiodactyla | *Balaenoptera acutorostrata* | DMK | 7.0000 | Sarcophagy | Aquatic |
|  | *Tursiops truncatus* | DMK | 5.2430 | Sarcophagy | Aquatic |
|  | *Orcinus orca* | DMK | 6.6335 | Sarcophagy | Aquatic |
|  | *Delphinapterus leucas* | DMK | 6.1335 | Sarcophagy | Aquatic |
|  | *Physeter catodon* | DMK | 7.1469 | Sarcophagy | Aquatic |
|  | *Monodon monoceros* | DMK | 5.9542 | Sarcophagy | Aquatic |
|  | *Globicephala melas* | DMK | 5.9031 | Sarcophagy | Aquatic |
|  | *Phocoena sinus* | DMK | 4.6284 | Sarcophagy | Aquatic |
|  | *Lagenorhynchus obliquidens* | DMK | 5.0792 | Sarcophagy | Aquatic |
|  | *Lipotes vexillifer* | DMK | 4.9217 | Sarcophagy | Aquatic |
|  | *Neophocaena phocaenoides* | DMK | 4.5119 | Sarcophagy | Aquatic |
|  | *Balaenoptera musculus* | DMK | 7.0000 | Sarcophagy | Aquatic |
|  | *Hippopotamus amphibius* | UK | 6.1515 | Phytophagous | Aquatic |
|  | *Bos taurus* | DMK | 5.9542 | Phytophagous | Terrestrial |
|  | *Bison bison* | DMK | 5.7629 | Phytophagous | Terrestrial |
|  | *Bos indicus* | DMK | 5.9542 | Phytophagous | Terrestrial |
|  | *Bubalus bubalis* | DMK | 5.9165 | Phytophagous | Aquatic |
|  | *Sus scrofa* | CMK | 4.8535 | - | Terrestrial |
|  | *Ovis aries* | UK | 4.6990 | Phytophagous | Terrestrial |
|  | *Camelus dromedarius* | UK | 5.9031 | Phytophagous | Terrestrial |
|  | *Camelus bactrianus* | UK | 5.6503 | Phytophagous | Terrestrial |
|  | *Capra hircus* | UK | 4.5250 | Phytophagous | Terrestrial |
| Carnivora | *Lutra lutra* | DMK | 3.8375 | Sarcophagy | Aquatic |
|  | *Enhydra lutris* | DMK | 4.3711 | Sarcophagy | Aquatic |
|  | *Ursus maritimus* | DMK | 5.5472 | Sarcophagy | Aquatic |
|  | *Ursus americanus* | DMK | 4.9998 | - | Terrestrial |
|  | *Ursus arctos* | DMK | 5.1444 | - | Terrestrial |
|  | *Phoca vitulina* | DMK | 5.0000 | Sarcophagy | Aquatic |
|  | *Leptonychotes weddellii* | DMK | 5.6021 | Sarcophagy | Aquatic |
|  | *Neomonachus schauinslandi* | DMK | 5.3483 | Sarcophagy | Aquatic |
|  | *Mirounga leonina* | DMK | 6.2041 | Sarcophagy | Aquatic |
|  | *Eumetopias jubatus* | DMK | 5.4914 | Sarcophagy | Aquatic |
|  | *Zalophus californianus* | DMK | 4.9031 | Sarcophagy | Aquatic |
|  | *Odobenus rosmarus* | DMK | 5.9165 | Sarcophagy | Aquatic |
|  | *Callorhinus ursinus* | DMK | 4.6545 | Sarcophagy | Aquatic |
|  | *Felis catus* | UK | 3.4558 | Sarcophagy | Terrestrial |
|  | *Canis lupus familiars* | UK | 4.3808 | - | Terrestrial |
|  | *Panthera pardus* | UK | 4.6580 | Sarcophagy | Terrestrial |
|  | *Panthera tigris* | UK | 5.2110 | Sarcophagy | Terrestrial |
| Perissodactyla | *Diceros bicornis* | DMK | 6.0721 | Phytophagous | Terrestrial |
|  | *Ceratotherium simum* | DMK | 6.4698 | Phytophagous | Terrestrial |
|  | *Equus caballus* | UK | 5.3979 | Phytophagous | Terrestrial |
|  | *Equus asinus* | UK | 5.5119 | Phytophagous | Terrestrial |
| Chiroptera | *Rhinolophus ferrumequinum* | UK | 1.2788 | Sarcophagy | Terrestrial |
| Eulipotyphla | *Sorex araneus* | UK | 0.7853 | Sarcophagy | Terrestrial |
|  | *Erinaceus europaeus* | UK | 2.8871 | - | Terrestrial |
| Rodentia | *Castor canadensis* | CMK | 4.3389 | Phytophagous | Aquatic |
|  | *Mus musculus* | UK | 1.2304 | - | Terrestrial |
|  | *Cavia porcellus* | UK | 2.8621 | Phytophagous | Terrestrial |
|  | *Heterocephalus glaber* | UK | 1.7404 | Phytophagous | Terrestrial |
| Primates | *Homo sapiens* | CMK | 4.7686 | - | Terrestrial |
|  | *Macaca mulatta* | UK | 3.5697 | - | Terrestrial |
|  | *Aotus nancymaae* | UK | 2.8965 | Phytophagous | Terrestrial |
|  | *Papio anubis* | UK | 4.2487 | - | Terrestrial |
| Proboscidea | *Loxodonta africana* | DMK | 6.5955 | Phytophagous | Terrestrial |
|  | *Elephas maximus* | DMK | 6.4346 | Phytophagous | Terrestrial |
| Sirenia | *Trichechus manatus* | CMK | 5.6368 | Phytophagous | Aquatic |
| Macroscelidea | *Elephantulus edwardii* | UK | 1.6812 | Sarcophagy | Terrestrial |
| Cingulata | *Dasypus novemcinctus* | UK | 3.6237 | Sarcophagy | Terrestrial |
| Marsupialia | *Phascolarctos cinereus* | UK | 4.0107 | Phytophagous | Terrestrial |
|  | *Monodelphis domestica* | UK | 1.8513 | - | Terrestrial |
| Monotremata | *Ornithorhynchus anatinus* | UK | 3.6180 | Sarcophagy | Aquatic |

DMK: discrete multirenculate kidney; CMK: compound multirenculate kidney; UK: unilobar kidney; Lg (Body mass): log_10_^body mass^; -: omnivory.

**Table S2. Model comparison of reconstructing the ancestral state of mammalian renal structures**

| **Model** | **AICc** | **AICc_wt** |
| --- | --- | --- |
| DEC | 116.6 | 4.00E-09 |
| DEC + J | 80.12 | 0.34 |
| DIVALIKE | 107.5 | 3.80E-07 |
| DIVALIKE + J | 80.14 | 0.33 |
| BAYAREALIKE | 140.1 | 3.10E-14 |
| BAYAREALIKE + J | 80.14 | 0.33 |

Note:

DEC: dispersal-extinction-cladogenesis

DEC + J: dispersal-extinction-cladogenesis + jump dispersal parameter

DIVALIKE: dispersal-vicariance analysis under ML

DIVALIKE + J: dispersal-vicariance analysis under ML + jump dispersal parameter

BAYAREALIKE: Bayesian approach with a large number of areas under ML

BAYAREALIKE + J: Bayesian approach with a large number of areas under ML + jump dispersal parameter

**Table S3 The probabilities of renal structural types induced in the nodes.**

| **Node** | **Phenotype & Probability** | | | | |
| --- | --- | --- | --- | --- | --- |
| 63 | C 100.000000 | BC 0.000000 | AC 0.000000 | B 0.000000 | A 0.000000 |
| 64 | C 100.000000 | BC 0.000000 | AC 0.000000 | B 0.000000 | A 0.000000 |
| 65 | C 100.000000 | AC 0.000000 | BC 0.000000 | A 0.000000 | B 0.000000 |
| 66 | C 100.000000 | AC 0.000000 | BC 0.000000 | A 0.000000 | B 0.000000 |
| 67 | A 100.000000 | AC 0.000000 | AB 0.000000 | C 0.000000 | B 0.000000 |
| 68 | A 100.000000 | AC 0.000000 | AB 0.000000 | C 0.000000 | B 0.000000 |
| 69 | A 100.000000 | AC 0.000000 | AB 0.000000 | C 0.000000 | B 0.000000 |
| 70 | A 100.000000 | AC 0.000000 | AB 0.000000 | C 0.000000 | B 0.000000 |
| 71 | A 100.000000 | AC 0.000000 | AB 0.000000 | C 0.000000 | B 0.000000 |
| 72 | A 100.000000 | AC 0.000000 | AB 0.000000 | C 0.000000 | B 0.000000 |
| 73 | A 100.000000 | AC 0.000000 | AB 0.000000 | C 0.000000 | B 0.000000 |
| 74 | A 100.000000 | AC 0.000000 | AB 0.000000 | C 0.000000 | B 0.000000 |
| 75 | A 100.000000 | AC 0.000000 | C 0.000000 | AB 0.000000 | B 0.000000 |
| 76 | A 100.000000 | AC 0.000000 | AB 0.000000 | C 0.000000 | B 0.000000 |
| 77 | A 100.000000 | AC 0.000000 | AB 0.000000 | C 0.000000 | B 0.000000 |
| 78 | A 100.000000 | AC 0.000000 | C 0.000000 | AB 0.000000 | B 0.000000 |
| 79 | C 95.219790 | A 4.779889 | C 0.000319 | BC 0.000000 | B 0.000000 |
| 80 | C 99.589680 | A 0.409926 | AC 0.000394 | BC 0.000000 | B 0.000000 |
| 81 | C 100.000000 | AC 0.000000 | BC 0.000000 | A 0.000000 | B 0.000000 |
| 82 | A 100.000000 | AC 0.000000 | AB 0.000000 | C 0.000000 | B 0.000000 |
| 83 | C 95.210930 | A 4.788174 | AC 0.000896 | BC 0.000000 | B 0.000000 |
| 84 | C 99.561390 | A 0.437114 | AC 0.001502 | BC 0.000000 | B 0.000000 |
| 85 | C 100.000000 | BC 0.000000 | AC 0.000000 | B 0.000000 | A 0.000000 |
| 86 | A 100.000000 | AC 0.000000 | AB 0.000000 | C 0.000000 | B 0.000000 |
| 87 | A 100.000000 | AC 0.000000 | AB 0.000000 | C 0.000000 | B 0.000000 |
| 88 | A 100.000000 | AC 0.000000 | AB 0.000000 | C 0.000000 | B 0.000000 |
| 89 | A 100.000000 | AC 0.000000 | AB 0.000000 | C 0.000000 | B 0.000000 |
| 90 | A 100.000000 | AC 0.000000 | AB 0.000000 | C 0.000000 | B 0.000000 |
| 91 | A 100.000000 | AC 0.000000 | AB 0.000000 | C 0.000000 | B 0.000000 |
| 92 | A 100.000000 | AC 0.000000 | AB 0.000000 | C 0.000000 | B 0.000000 |
| 93 | A 100.000000 | AC 0.000000 | AB 0.000000 | C 0.000000 | B 0.000000 |
| 94 | A 100.000000 | AC 0.000000 | AB 0.000000 | C 0.000000 | B 0.000000 |
| 95 | A 100.000000 | AC 0.000000 | AB 0.000000 | C 0.000000 | B 0.000000 |
| 96 | A 100.000000 | AC 0.000000 | C 0.000000 | AB 0.000000 | B 0.000000 |
| 97 | C 88.081950 | A 11.918000 | AC 0.000057 | BC 0.000000 | B 0.000000 |
| 98 | A 100.000000 | AC 0.000000 | AB 0.000000 | C 0.000000 | B 0.000000 |
| 99 | A 100.000000 | AC 0.000000 | AB 0.000000 | C 0.000000 | B 0.000000 |
| 100 | A 100.00000 | AC 0.00000 | AB 0.00000 | C 0.00000 | B 0.00000 |
| 101 | C 100.00000 | AC 0.00000 | BC 0.00000 | A 0.00000 | B 0.00000 |
| 102 | C 88.081950 | A 11.918000 | AC 0.000057 | BC 0.00000 | B 0.00000 |
| 103 | C 91.577270 | A 8.4226020 | AC 0.000127 | BC 0.000000 | B 0.000000 |
| 104 | C 87.372890 | B 8.407232 | A 4.218986 | BC 0.000765 | AC 0.000127 |
| 105 | C 99.402210 | B 0.387327 | A 0.209019 | BC 0.001126 | AC 0.000322 |
| 106 | C 99.931240 | A 0.046388 | B 0.019202 | AC 0.001958 | BC 0.001211 |
| 107 | C 99.993730 | A 0.002142 | AC 0.002010 | BC 0.001233 | B 0.000888 |
| 108 | C 99.996600 | AC 0.002023 | BC 0.001238 | A 0.000102 | B 0.000043 |
| 109 | C 100.00000 | BC 0.00000 | AC 0.00000 | B 0.00000 | A 0.00000 |
| 110 | C 95.372290 | B 4.604794 | BC 0.022914 | AC 0.000000 | A 0.000000 |
| 111 | C 99.749180 | B 0.222496 | BC 0.028327 | AC 0.000000 | AB 0.00000 |
| 112 | C 100.00000 | BC 0.00000 | AC 0.00000 | B 0.00000 | A 0.00000 |
| 113 | C 95.372290 | B 4.604794 | BC 0.022914 | AC 0.000000 | A 0.000000 |
| 114 | C 99.749180 | B 0.222496 | BC 0.028327 | AC 0.000000 | AB 0.00000 |
| 115 | C 99.911130 | BC 0.059102 | B 0.029763 | AC 0.000000 | AB 0.00000 |
| 116 | C 99.935060 | BC 0.061197 | AC 0.002026 | B 0.001718 | A 0.000006 |
| 117 | A 100.00000 | AC 0.00000 | AB 0.00000 | B 0.00000 | C 0.00000 |
| 118 | B 50.000680 | A 49.999320 | AB 0.000000 | BC 0.00000 | AC 0.0000 |
| 119 | C 94.696350 | B 2.366976 | A 2.365696 | BC 0.285528 | AC 0.285454 |
| 120 | C 99.042210 | BC 0.352972 | AC 0.352880 | B 0.126600 | A 0.125337 |
| 121 | C 99.179410 | BC 0.430293 | AC 0.370833 | B 0.009824 | A 0.009647 |
| 122 | C 99.188640 | BC 0.434125 | AC 0.374596 | B 0.001333 | A 0.001309 |
| 123 | C 99.189060 | BC 0.435030 | AC 0.375485 | B 0.000214 | A 0.000210 |

A: discrete multirenculate kidney; B: compound multirenculate kidney; C: unilobar kidney.

**Table S4. Genes under rapid evolution in DMK mammals identified using branch model.**

| **Gene** | **M0: one-ratio** | **M2: two-ratio** | | **2ΔlnL** | **Adjusted**  **p value** |
| --- | --- | --- | --- | --- | --- |
|  | **ω** | **ω 0** | **ω 1** |  |  |
| *DYNC2H1* | 0.07959 | 0.07342 | 0.17061 | 186.1796 | 0 |
| *DNAH11* | 0.18058 | 0.17077 | 0.31091 | 144.5654 | 0 |
| *DNAH5* | 0.11751 | 0.11145 | 0.19898 | 121.8492 | 0 |
| *CTNNB1* | 0.00331 | 0.00166 | 0.04001 | 50.68641 | 1.11E-11 |
| *ZBTB14* | 0.01099 | 0.0091 | 0.05175 | 35.33501 | 2.28E-08 |
| *PCNT* | 0.25267 | 0.24618 | 0.33067 | 30.274 | 2.56E-07 |
| *TBC1D32* | 0.19455 | 0.18695 | 0.32439 | 28.22369 | 6.33E-07 |
| *ADAMTS18* | 0.13562 | 0.13037 | 0.21391 | 22.75619 | 9.43E-06 |
| *CEP290* | 0.14508 | 0.14121 | 0.20733 | 19.1302 | 5.56E-05 |
| *CPLANE1* | 0.37108 | 0.3657 | 0.44692 | 11.59877 | 0.002706 |
| *CC2D2A* | 0.1035 | 0.10083 | 0.14395 | 7.427974 | 0.023936 |
| *MKS1* | 0.12543 | 0.1212 | 0.18506 | 7.123846 | 0.025989 |
| *FAT4* | 0.07263 | 0.07158 | 0.08601 | 6.926622 | 0.026783 |
| *BMP4* | 0.06481 | 0.06143 | 0.1325 | 6.713622 | 0.02802 |

**Table S5. Genes under rapid evolution in Non-DMK mammals identified using branch model.**

| **Gene** | **M0: one-ratio** | **M2: two-ratio** | | **2ΔlnL** | **Adjusted**  **p value** |
| --- | --- | --- | --- | --- | --- |
|  | **ω** | **ω 0** | **ω 1** |  |  |
| *FAT4* | 0.07263 | 0.05794 | 0.09164 | 138.480034 | 0.0000 |
| *CYFIP2* | 0.0054 | 0.00116 | 0.00852 | 33.469128 | 0.0000 |
| *SLIT2* | 0.03731 | 0.02706 | 0.04432 | 23.30748 | 0.0000 |
| *TBC1D32* | 0.19455 | 0.17584 | 0.21234 | 11.386204 | 0.0037 |
| *TP53* | 0.17945 | 0.15186 | 0.20793 | 11.508628 | 0.0037 |
| *NFIA* | 0.01038 | 0.0058 | 0.01686 | 9.128352 | 0.0087 |
| *GATA3* | 0.01951 | 0.01398 | 0.02442 | 8.255436 | 0.0131 |
| *PTPRS* | 0.02189 | 0.01959 | 0.02393 | 7.645694 | 0.0171 |
| *CXCR4* | 0.06179 | 0.04895 | 0.07392 | 7.207238 | 0.0204 |
| *FOXC1* | 0.02868 | 0.02338 | 0.03376 | 6.436526 | 0.0291 |
| *LHX1* | 0.00959 | 0.00507 | 0.01365 | 6.362284 | 0.0291 |

**Table S6 GO enrichment of rapidly evolving genes in DMK mammals (gene count≥3)**

| **ID** | **Description** | **GeneRatio** | **BgRatio** | **p.adjust** | **qvalue** | **Count** |
| --- | --- | --- | --- | --- | --- | --- |
| **Biological Process (BP)** | | | | | | |
| GO:0060271 | cilium assembly | 5/12 | 211/11526 | 0.000201633 | 9.66E-05 | 5 |
| GO:0044782 | cilium organization | 5/12 | 222/11526 | 0.000201633 | 9.66E-05 | 5 |
| GO:0001822 | kidney development | 5/12 | 252/11526 | 0.000240408 | 0.000115129 | 5 |
| GO:0072001 | renal system development | 5/12 | 257/11526 | 0.000240408 | 0.000115129 | 5 |
| GO:0001655 | urogenital system development | 5/12 | 291/11526 | 0.000343159 | 0.000164335 | 5 |
| GO:0120031 | plasma membrane bounded cell projection assembly | 5/12 | 370/11526 | 0.000827176 | 0.000396126 | 5 |
| GO:0030031 | cell projection assembly | 5/12 | 378/11526 | 0.000880585 | 0.000421703 | 5 |
| GO:0007507 | heart development | 5/12 | 471/11526 | 0.001938877 | 0.000928508 | 5 |
| GO:0048598 | embryonic morphogenesis | 5/12 | 472/11526 | 0.001938877 | 0.000928508 | 5 |
| GO:0009953 | dorsal/ventral pattern formation | 4/12 | 63/11526 | 0.000201633 | 9.66E-05 | 4 |
| GO:0009799 | specification of symmetry | 4/12 | 88/11526 | 0.000201633 | 9.66E-05 | 4 |
| GO:0030326 | embryonic limb morphogenesis | 4/12 | 97/11526 | 0.000201633 | 9.66E-05 | 4 |
| GO:0035113 | embryonic appendage morphogenesis | 4/12 | 97/11526 | 0.000201633 | 9.66E-05 | 4 |
| GO:0072175 | epithelial tube formation | 4/12 | 102/11526 | 0.000224191 | 0.000107363 | 4 |
| GO:0035107 | appendage morphogenesis | 4/12 | 113/11526 | 0.000240408 | 0.000115129 | 4 |
| GO:0035108 | limb morphogenesis | 4/12 | 113/11526 | 0.000240408 | 0.000115129 | 4 |
| GO:0035148 | tube formation | 4/12 | 114/11526 | 0.000240408 | 0.000115129 | 4 |
| GO:0048736 | appendage development | 4/12 | 138/11526 | 0.000411302 | 0.000196968 | 4 |
| GO:0060173 | limb development | 4/12 | 138/11526 | 0.000411302 | 0.000196968 | 4 |
| GO:0003002 | regionalization | 4/12 | 254/11526 | 0.00237525 | 0.001137482 | 4 |
| GO:0060562 | epithelial tube morphogenesis | 4/12 | 262/11526 | 0.002391918 | 0.001145464 | 4 |
| GO:0007389 | pattern specification process | 4/12 | 326/11526 | 0.004076318 | 0.001952106 | 4 |
| GO:0048568 | embryonic organ development | 4/12 | 346/11526 | 0.00449932 | 0.002154677 | 4 |
| GO:0002009 | morphogenesis of an epithelium | 4/12 | 401/11526 | 0.006272113 | 0.003003649 | 4 |
| GO:0007423 | sensory organ development | 4/12 | 435/11526 | 0.007954666 | 0.003809406 | 4 |
| GO:1903827 | regulation of cellular protein localization | 4/12 | 440/11526 | 0.008121956 | 0.003889519 | 4 |
| GO:0048729 | tissue morphogenesis | 4/12 | 491/11526 | 0.010618479 | 0.005085079 | 4 |
| GO:2000136 | regulation of cell proliferation involved in heart morphogenesis | 3/12 | 17/11526 | 0.000201633 | 9.66E-05 | 3 |
| GO:0061323 | cell proliferation involved in heart morphogenesis | 3/12 | 18/11526 | 0.000201633 | 9.66E-05 | 3 |
| GO:0061311 | cell surface receptor signaling pathway involved in heart development | 3/12 | 23/11526 | 0.000201633 | 9.66E-05 | 3 |
| GO:0060914 | heart formation | 3/12 | 25/11526 | 0.000201633 | 9.66E-05 | 3 |
| GO:0060976 | coronary vasculature development | 3/12 | 35/11526 | 0.000296487 | 0.000141985 | 3 |
| GO:1905515 | non-motile cilium assembly | 3/12 | 43/11526 | 0.000450442 | 0.000215712 | 3 |
| GO:0042733 | embryonic digit morphogenesis | 3/12 | 45/11526 | 0.000493807 | 0.000236479 | 3 |
| GO:0048645 | animal organ formation | 3/12 | 54/11526 | 0.000821293 | 0.000393309 | 3 |
| GO:0003279 | cardiac septum development | 3/12 | 87/11526 | 0.00219924 | 0.001053193 | 3 |
| GO:0009855 | determination of bilateral symmetry | 3/12 | 87/11526 | 0.00219924 | 0.001053193 | 3 |
| GO:0001838 | embryonic epithelial tube formation | 3/12 | 92/11526 | 0.002391918 | 0.001145464 | 3 |
| GO:2000027 | regulation of animal organ morphogenesis | 3/12 | 96/11526 | 0.002391918 | 0.001145464 | 3 |
| GO:0048706 | embryonic skeletal system development | 3/12 | 99/11526 | 0.002529017 | 0.00121112 | 3 |
| GO:0016331 | morphogenesis of embryonic epithelium | 3/12 | 114/11526 | 0.003273823 | 0.001567799 | 3 |
| GO:0048592 | eye morphogenesis | 3/12 | 128/11526 | 0.004118357 | 0.001972238 | 3 |
| GO:0048754 | branching morphogenesis of an epithelial tube | 3/12 | 130/11526 | 0.004118357 | 0.001972238 | 3 |
| GO:0003205 | cardiac chamber development | 3/12 | 140/11526 | 0.004676506 | 0.00223953 | 3 |
| GO:0048839 | inner ear development | 3/12 | 141/11526 | 0.004676506 | 0.00223953 | 3 |
| GO:0061138 | morphogenesis of a branching epithelium | 3/12 | 158/11526 | 0.005789684 | 0.002772619 | 3 |
| GO:0043583 | ear development | 3/12 | 160/11526 | 0.005845955 | 0.002799567 | 3 |
| GO:0001763 | morphogenesis of a branching structure | 3/12 | 169/11526 | 0.006696179 | 0.00320673 | 3 |
| GO:0033157 | regulation of intracellular protein transport | 3/12 | 197/11526 | 0.008965232 | 0.004293356 | 3 |
| GO:0090596 | sensory organ morphogenesis | 3/12 | 202/11526 | 0.009429592 | 0.004515733 | 3 |
| GO:0003007 | heart morphogenesis | 3/12 | 204/11526 | 0.009530516 | 0.004564064 | 3 |
| GO:0048562 | embryonic organ morphogenesis | 3/12 | 223/11526 | 0.011429613 | 0.005473522 | 3 |
| GO:0043010 | camera-type eye development | 3/12 | 267/11526 | 0.016463674 | 0.007884281 | 3 |
| GO:0032386 | regulation of intracellular transport | 3/12 | 278/11526 | 0.017644348 | 0.008449693 | 3 |
| GO:0001654 | eye development | 3/12 | 301/11526 | 0.020294634 | 0.009718888 | 3 |
| GO:0150063 | visual system development | 3/12 | 304/11526 | 0.020590483 | 0.009860567 | 3 |
| GO:0048880 | sensory system development | 3/12 | 309/11526 | 0.021418194 | 0.010256949 | 3 |
| GO:0030900 | forebrain development | 3/12 | 313/11526 | 0.02191679 | 0.010495721 | 3 |
| GO:0001501 | skeletal system development | 3/12 | 419/11526 | 0.043966022 | 0.021054867 | 3 |
| GO:1905114 | cell surface receptor signaling pathway involved in cell-cell signaling | 3/12 | 421/11526 | 0.044286775 | 0.021208473 | 3 |
| GO:0051223 | regulation of protein transport | 3/12 | 424/11526 | 0.044640065 | 0.02137766 | 3 |
| GO:0070201 | regulation of establishment of protein localization | 3/12 | 445/11526 | 0.048878934 | 0.02340761 | 3 |
| **Cellular Component (CC)** | | | | | | |
| GO:0005929 | cilium | 4/12 | 410/11853 | 0.017169457 | 0.012769047 | 4 |
| GO:0005813 | centrosome | 4/12 | 472/11853 | 0.021899781 | 0.016287023 | 4 |
| GO:0035869 | ciliary transition zone | 3/12 | 52/11853 | 0.001567636 | 0.001165862 | 3 |
| GO:0005814 | centriole | 3/12 | 96/11853 | 0.004941788 | 0.003675243 | 3 |

**Table S7 KEGG enrichment of rapidly evolving genes in DMK mammals.**

| **ID** | **Description** | **GeneRatio** | **BgRatio** | **p.adjust** | **qvalue** | **Count** |
| --- | --- | --- | --- | --- | --- | --- |
| hsa05022 | Pathways of neurodegeneration - multiple diseases | 3/5 | 476/8112 | 0.021457845 | 0.01317587 | 3 |
| hsa05217 | Basal cell carcinoma | 2/5 | 63/8112 | 0.021051659 | 0.012926458 | 2 |
| hsa04919 | Thyroid hormone signaling pathway | 2/5 | 121/8112 | 0.021457845 | 0.01317587 | 2 |
| hsa05418 | Fluid shear stress and atherosclerosis | 2/5 | 139/8112 | 0.021457845 | 0.01317587 | 2 |
| hsa04550 | Signaling pathways regulating pluripotency of stem cells | 2/5 | 143/8112 | 0.021457845 | 0.01317587 | 2 |
| hsa04390 | Hippo signaling pathway | 2/5 | 157/8112 | 0.021492723 | 0.013197286 | 2 |
| hsa05132 | Salmonella infection | 2/5 | 249/8112 | 0.04539359 | 0.027873257 | 2 |

**Table S8 GO and KEGG enrichment of positively selected genes in DMK mammals (gene count≥3)**

| **ID** | **Description** | **GeneRatio** | **BgRatio** | **p.adjust** | **qvalue** | **Count** |
| --- | --- | --- | --- | --- | --- | --- |
| **GO enrichment** | | | | | | |
| GO:0060562 | epithelial tube morphogenesis | 4/6 | 262/11526 | 0.001216 | 0.000469 | 4 |
| GO:0050767 | regulation of neurogenesis | 4/6 | 305/11526 | 0.001216 | 0.000469 | 4 |
| GO:0051960 | regulation of nervous system development | 4/6 | 364/11526 | 0.001216 | 0.000469 | 4 |
| GO:0002009 | morphogenesis of an epithelium | 4/6 | 401/11526 | 0.001338 | 0.000516 | 4 |
| GO:0060284 | regulation of cell development | 4/6 | 424/11526 | 0.001444 | 0.000557 | 4 |
| GO:0048729 | tissue morphogenesis | 4/6 | 491/11526 | 0.001702 | 0.000656 | 4 |
| GO:0001838 | embryonic epithelial tube formation | 3/6 | 92/11526 | 0.001216 | 0.000469 | 3 |
| GO:0021987 | cerebral cortex development | 3/6 | 99/11526 | 0.001216 | 0.000469 | 3 |
| GO:0072175 | epithelial tube formation | 3/6 | 102/11526 | 0.001216 | 0.000469 | 3 |
| GO:0016331 | morphogenesis of embryonic epithelium | 3/6 | 114/11526 | 0.001315 | 0.000507 | 3 |
| GO:0035148 | tube formation | 3/6 | 114/11526 | 0.001315 | 0.000507 | 3 |
| GO:0048754 | branching morphogenesis of an epithelial tube | 3/6 | 130/11526 | 0.001444 | 0.000557 | 3 |
| GO:0050770 | regulation of axonogenesis | 3/6 | 136/11526 | 0.001444 | 0.000557 | 3 |
| GO:0021543 | pallium development | 3/6 | 144/11526 | 0.001457 | 0.000561 | 3 |
| GO:1990138 | neuron projection extension | 3/6 | 144/11526 | 0.001457 | 0.000561 | 3 |
| GO:0061138 | morphogenesis of a branching epithelium | 3/6 | 158/11526 | 0.001748 | 0.000673 | 3 |
| GO:0001763 | morphogenesis of a branching structure | 3/6 | 169/11526 | 0.001959 | 0.000755 | 3 |
| GO:0048588 | developmental cell growth | 3/6 | 195/11526 | 0.002612 | 0.001006 | 3 |
| GO:0050769 | positive regulation of neurogenesis | 3/6 | 195/11526 | 0.002612 | 0.001006 | 3 |
| GO:0060560 | developmental growth involved in morphogenesis | 3/6 | 196/11526 | 0.002612 | 0.001006 | 3 |
| GO:0021537 | telencephalon development | 3/6 | 201/11526 | 0.002718 | 0.001047 | 3 |
| GO:0003007 | heart morphogenesis | 3/6 | 204/11526 | 0.002746 | 0.001058 | 3 |
| GO:0051962 | positive regulation of nervous system development | 3/6 | 227/11526 | 0.003536 | 0.001362 | 3 |
| GO:0010720 | positive regulation of cell development | 3/6 | 262/11526 | 0.004959 | 0.001911 | 3 |
| GO:0030900 | forebrain development | 3/6 | 313/11526 | 0.007504 | 0.002892 | 3 |
| GO:0034329 | cell junction assembly | 3/6 | 332/11526 | 0.008096 | 0.00312 | 3 |
| GO:0050808 | synapse organization | 3/6 | 332/11526 | 0.008096 | 0.00312 | 3 |
| GO:0007409 | axonogenesis | 3/6 | 346/11526 | 0.008891 | 0.003426 | 3 |
| GO:0010975 | regulation of neuron projection development | 3/6 | 375/11526 | 0.010161 | 0.003916 | 3 |
| GO:0061564 | axon development | 3/6 | 385/11526 | 0.010368 | 0.003995 | 3 |
| GO:0016049 | cell growth | 3/6 | 398/11526 | 0.010586 | 0.004079 | 3 |
| GO:0048667 | cell morphogenesis involved in neuron differentiation | 3/6 | 445/11526 | 0.012836 | 0.004946 | 3 |
| GO:0007507 | heart development | 3/6 | 471/11526 | 0.014344 | 0.005527 | 3 |
| GO:0048598 | embryonic morphogenesis | 3/6 | 472/11526 | 0.014344 | 0.005527 | 3 |
| GO:0043009 | chordate embryonic development | 3/6 | 487/11526 | 0.014958 | 0.005764 | 3 |
| GO:0009792 | embryo development ending in birth or egg hatching | 3/6 | 500/11526 | 0.015571 | 0.006 | 3 |
| **KEGG enrichment** | | | | | | |
| hsa04360 | Axon guidance | 3/4 | 182/8112 | 0.001355098 | 0.000966284 | 3 |

**Table S9 List of 45 genes related to duplex/multiplex kidney formation used in this study.**

| **Source** | **Gene list** |
| --- | --- |
| MGI database | *CPLANE1, CXCR4, CYFIP2, DNAH5, DNAH11, FOXC2, GEN1, LZTS2, PCNT, PLXNB2, PTPRS, PTPRF, WDPCP, ZBTB14* |
| Article | *GDNF, ROBO2, SLIT2, FOXC1, SOX11, BMP4, HSPB11, IFT27, GLI3, AGTR2, TP53, FAT4, FJX1, HOXB7, CTNNB1, SPRY1, GATA3, WNT5A, ROR2, NCAM1, PAX2, LHX1, CC2D2A, MKS1, CEP290, DYNC2H1, TBC1D32, TMEM67, SOX17, NFIA, ADAMTS18* |
